# Autism subtypes identified using cross-species functional connectivity analyses

**DOI:** 10.1038/s41593-026-02287-z

**Published:** 2026-05-15

**Authors:** Marco Pagani, Valerio Zerbi, Silvia Gini, Filomena Grazia Alvino, Abhishek Banerjee, Andrea Barberis, M. Albert Basson, Yuri Bozzi, Alberto Galbusera, Jacob Ellegood, Michela Fagiolini, Jason P. Lerch, Michela Matteoli, Caterina Montani, Davide Pozzi, Giovanni Provenzano, Maria Luisa Scattoni, Nicole Wenderoth, Ting Xu, Michael V. Lombardo, Michael P. Milham, Adriana Di Martino, Alessandro Gozzi

**Affiliations:** 1 Functional Neuroimaging Laboratory, Istituto Italiano di Tecnologia, Center for Neuroscience and Cognitive Systems, CNCS@UNITN, Rovereto, Italy; 2 Autism Center, Child Mind Institute, New York, NY USA; 3 IMT School for Advanced Studies, Lucca, Italy; 4 Department of Psychiatry, University of Geneva, Geneva, Switzerland; 5 Department of Basic Neurosciences, University of Geneva, Geneva, Switzerland; 6 Center for Mind and Brain Sciences (CIMeC), University of Trento, Rovereto, Italy; 7 Brain Research Institute, University of Zurich, Zurich, Switzerland; 8 Adaptive Decisions Lab, Blizard Institute, Queen Mary University of London, London, UK; 9 Department of Pharmacology, University of Oxford, Oxford, UK; 10 Synaptic Plasticity of Inhibitory Networks, Istituto Italiano di Tecnologia, Genova, Italy; 11 Centre for Craniofacial and Regenerative Biology, King’s College London, London, UK; 12 Department of Clinical and Biomedical Sciences, University of Exeter, Exeter, UK; 13 Bloorview Research Institute, Holland Bloorview Kids Rehabilitation Hospital, Toronto, Ontario Canada; 14 Boston Children’s Hospital, Harvard Medical School, Boston, MA USA; 15 International Research Center for Neurointelligence, University of Tokyo, Tokyo, Japan; 16 Oxford Centre for Integrative Neuroimaging, FMRIB, Nuffield Department of Clinical Neurosciences, University of Oxford, Oxford, UK; 17 Mouse Imaging Centre, The Hospital for Sick Children, Toronto, Ontario Canada; 18 Department of Medical Biophysics, University of Toronto, Toronto, Ontario Canada; 19 Department of Biomedical Sciences, Humanitas University, Milan, Italy; 20 IRCCS Humanitas Research Hospital, Milan, Italy; 21 Department of Cellular, Computational and Integrative Biology. University of Trento, Trento, Italy; 22 National Center for Rare Diseases, Istituto Superiore di Sanità, Rome, Italy; 23 Neural Control of Movement Lab, ETH Zürich, Zurich, Switzerland; 24 Center for Integrative Developmental Neuroscience, Child Mind Institute, New York, NY USA; 25 Laboratory for Autism and Neurodevelopmental Disorders, Istituto Italiano di Tecnologia, Center for Neuroscience and Cognitive Systems, Rovereto, Italy; 26 Center for Biomedical Imaging and Neuromodulation, Nathan S. Kline Institute for Psychiatric Research, Orangeburg, NY USA; 27 Present Address: IRCCS Ospedale Policlinico San Martino, Genova, Italy

**Keywords:** Autism spectrum disorders, Functional magnetic resonance imaging

## Abstract

It is often assumed that phenotypic heterogeneity in autism reflects underlying pathobiological variation. However, direct evidence supporting this link is lacking. Leveraging cross-species functional neuroimaging, we show that brain dysconnectivity patterns in autism can be parsed into biologically dissociable subtypes. Specifically, we found that functional magnetic resonance imaging (fMRI) connectivity alterations in 20 distinct genetic mouse models of autism cluster into hypoconnectivity-dominant and hyperconnectivity-dominant subtypes. These subtypes are linked to distinct biological pathways, with hypoconnectivity being associated with synaptic dysfunction and hyperconnectivity reflecting transcriptional and immune-related alterations. Here we identified analogous hypoconnectivity and hyperconnectivity subtypes in a multicenter human fMRI dataset of *n* = 940 individuals with idiopathic autism and *n* = 1,036 neurotypical individuals. The human autism subtypes are highly replicable, are associated with distinct functional network architectures and behavioral profiles and recapitulate the synaptic and immune-related pathways identified in the rodent dataset. Our work provides a new empirical framework for targeted subtyping of the autism spectrum.

## Abstract (web summary)

Pagani et al. used cross-species fMRI to reveal two autism subtypes, characterized by lower and higher brain connectivity and linked to synaptic and immune-related pathways, respectively.

## Main

Autism spectrum disorder (ASD, hereafter referred to as autism) is characterized by highly heterogeneous phenotypic presentation, encompassing variable expression of core diagnostic symptoms and associated features, such as language, intellectual, motor and adaptive functioning^1–3^. This variability also manifests in multiple neuroimaging endophenotypes, including differences in brain activation patterns, functional connectivity and morphometric features^3–8^. Such heterogeneity has long been recognized in the neuroimaging literature, with reviews highlighting variability in methodological approaches and developmental effects on brain connectivity^9,10^ as well as broader issues of reproducibility in autism neuroimaging^11^. Recent investigations have revealed a similarly striking heterogeneity in the genetic and biological processes known to be associated with autism^12–14^. Specifically, large-scale genetic studies have shown that the high heritability of autism involves multiple (>100) rare, highly penetrant mutations, as well as common genetic risk variants^12–14^. These genetic factors, alone or in combination, affect highly heterogeneous biological pathways, including synaptic activity, neurogenesis, cell migration and gene transcription^15,16^. Beyond genetics, environmental influences, particularly prenatal inflammatory conditions and immune dysfunction, have also been shown to modulate autism risk^17^.

A common assumption exists that the phenotypic heterogeneity observed in autism directly reflects underlying pathobiological heterogeneity^18–21^. However, causal evidence in support of this hypothesis is lacking. Current genetic analyses do not allow for a reliable biological stratification of autism, because only 20% of individuals harbor clinically pathogenic rare variants, and no single mutation accounts for more than 1% of cases^14^. Given these limitations, recent efforts have focused on identifying phenotypically defined subtypes within autistic populations. Statistical clustering of clinical and neuroimaging phenotypes has been used to identify putative autism subtypes—that is, subgroups of individuals with autism characterized by more uniform clinical and/or neural phenotypes, putatively representing different pathobiological mechanisms^19–22^. However, despite the potential of this approach, direct evidence of its validity remains elusive. Most subtyping studies to date lack plausible neurobiological validation and rely, at best, on tentative associations between neuroimaging metrics and normative gene expression patterns^18,21^.

Cross-species approaches enabling the biological decoding of autism-relevant phenotypes^23^ could bridge this critical knowledge gap, offering a pivotal avenue for advancing autism research. Rodent models offer a unique experimental approach to isolate and probe the effect of autism-relevant etiological factors on brain connectivity, with minimal genetic or environmental confounds^23,24^. Leveraging technical advances in cross-species functional neuroimaging, we and others have highlighted remarkably conserved fMRI connectivity alterations in both clinical populations and mouse lines harboring corresponding autism-relevant genetic variants^25–28^. Large-scale functional neuroimaging across multiple mouse autism models thus offers an unprecedented opportunity to biologically decode autism heterogeneity into etiologically distinct dysconnectivity signatures and to potentially guide cross-species subtyping. Within this translational framework, rodent models have proven instrumental in modeling key autism-relevant immune alterations, as well as many of the multiple high-penetrance, rare genetic variants that constitute approximately 20% of autism’s genetic architecture^29^. Importantly, postmortem studies have highlighted substantial overlap between pathways dysregulated in idiopathic autism and those affected by rare genetic variants, further validating the use of rodent models to probe autism-relevant pathobiological mechanisms^16,30^.

Building upon this notion, we reason that cross-species fMRI map decoding^23,28,31^ could empirically guide the identification of brain dysconnectivity subtypes in autism, reflecting some of the biological pathways modeled in rodents. Using this approach, here we show that variability in brain functional connectivity encodes dissociable biological pathways. Specifically, we report that fMRI connectivity alterations in 20 mouse models of autism can be clustered into two dominant hypoconnectivity and hyperconnectivity subtypes reflecting synaptic and transcriptional/immune-related pathways, respectively. Guided by these rodent findings, we identified analogous hypoconnectivity and hyperconnectivity subtypes in fMRI scans from individuals with an autism diagnosis and link these patterns to autism-relevant synaptic and immune mechanisms. Our work documents that heterogeneous fMRI connectivity in idiopathic autism encodes for dissociable pathobiological mechanisms, laying a foundation for biologically informed clinical subtyping of the autism spectrum.

## Results

### fMRI dysconnectivity in 20 autism mouse models clusters into dominant hypoconnectivity and hyperconnectivity subtypes

Previous investigations using resting-state fMRI revealed highly heterogeneous patterns of atypical connectivity (here termed fMRI dysconnectivity) in individuals with autism^32,33^. Although this heterogeneity is often assumed to reflect underlying etiopathological variation, direct evidence in support of this hypothesis is lacking. To empirically probe this notion, we examined resting-state fMRI connectivity in an aggregated database of 20 different mouse lines modeling autism-relevant genetic mutations spanning different pathways (for example, synaptic mechanisms, protein translation, transcriptional regulation and chromatin remodeling) as well as immune-related mechanisms known to be relevant to autism. We hypothesized that if biological heterogeneity markedly contributes to the phenotypic variability observed with fMRI, dysconnectivity clusters should be associated with distinct etiological mechanisms as indicated by their gene mutations and the affected transcriptional pathways. By using a relatively large number of different mouse models, we aimed to capture the diverse etiological landscape of autism, enabling us to empirically test our hypothesis.

Table 1 provides detailed information on the employed mouse model database, which represents an extension of a previously described dataset^34^. Notably, each scanned model contains a control group of wild-type control littermates. This allowed us to precisely probe the fMRI connectivity alterations characterizing different pathobiological mechanisms with negligible environmental and genetic confounds. To facilitate cross-species translation of mouse findings to human populations, connectivity differences associated with each etiology were mapped at the voxel level using weighted degree centrality. This metric quantifies the mean fMRI connectivity of each voxel^35^ and previously revealed similar brain dysconnectivity signatures in rodents and humans harboring syntenic autism risk^26–28^.

**Table 1:** Autism-related mouse models

Scanning site	Mouse model	Gene/etiology modeled	SFARI score^a^	Mutants versus controls (*n*)	Females	Age range (weeks)	Reference
IIT	*Shank3B*^−/−^	*Shank3*	1S	10 vs 11	0 vs 0	19–21	^47^
IIT	*Cntnap2*^−*/*−^	*Cntnap2*	2S	13 vs 13	0 vs 0	12–16	^74^
IIT	16p11.2^+/−^	16p11.2 deletion	CNV	12 vs 11	0 vs 0	12	^27^
IIT	22q11.2^+/−^	22q11.2 deletion	CNV	22 vs 22	11 vs 8	15–17	^28^
IIT	*Syn2*^−*/*−^	*Syn2*	2	10 vs 10	0 vs 0	30–31	^75^
IIT	BTBR ^T+tpr3tf/J^	Callosal agenesis	-	10 vs 10	0 vs 0	26	^76^
IIT	*Chd8*^+/−^	*Chd8*	1S	19 vs 23	13 vs 14	15–17	^77^
IIT	*Oxtr*^−^^/^^−^	*Oxtr*	2	13 vs 15	0 vs 0	17–34	^78^
IIT	*Tsc2*^+/−^	*Tsc2*	1S	20 vs 20	0 vs 0	4	^26^
IIT	*Ube3A*^2X^	*Ube3a*	1S	20 vs 20	10 vs 10	11–29	^79^
IIT	*Nlgn*^−*/*−^	*Nlgn3*	1	13 vs 18	0 vs 0	8–20	^80^
IIT	*Nlgn* ^*KI*^	*Nlgn3-r451c*	-	15 vs 16	0 vs 0	26–35	^81^
ETH	*Cdkl5*^−*/*−^	*Cdkl5*	1S	10 vs 9	0 vs 0	23–25	^82^
ETH	*Cdkl5*^+/−^	*Cdkl5*	1S	11 vs 10	11 vs 10	17–19	^82^
ETH	*En2*^−*/*−^	*En2*	2	8 vs 8	5 vs 4	12–16	^83^
ETH	*Fmr1*^−*/y*^	*Fmr1*	1S	21 vs 19	0 vs 0	13–16	^84^
ETH	*Mecp2*^+/−^	*Mecp2*	1S	13 vs 10	13 vs 10	12–14	^85^
ETH	*Sgsh*^−*/*−^	*Sgsh*	S	14 vs 16	0 vs 0	20–22	^86^
ETH	IL-6	Immune/environmental	-	9 vs 7	0 vs 0	14	^60^
ETH	*Trem2*^−*/*−^	*Trem2*	-	10 vs 8	4 vs 4	12–15	^61^

^a^ SFARI scores were derived from https://gene.sfari.org/ in January 2025 and indicate the strength of the evidence of their implications in autism on a scale ranging from 1 (high) to 3 (low).

CNV, copy number variant; S, syndromic; SFARI, Simons Foundation Autism Research Initiative; vs, versus.

**Fig. 1:**
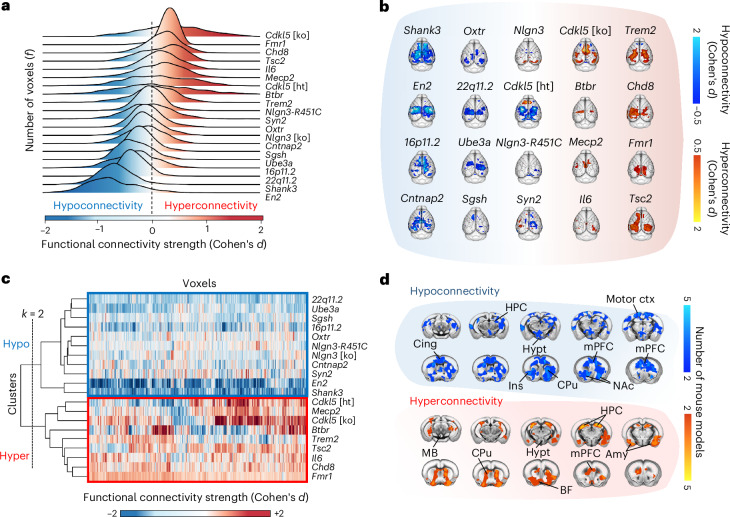
fMRI connectivity in 20 autism mouse models clusters into dominant hypoconnectivity and hyperconnectivity subtypes. **a**, Ridgeline plot quantifications of whole-brain fMRI connectivity differences (mutant versus control) for the 20 autism-relevant mouse models examined. Ridgelines show voxel distribution of fMRI connectivity strength indexed by Cohen’s *d* color coded by connectivity difference (blue: lower connectivity in mouse mutants versus control—that is, hypoconnectivity; red: higher connectivity in mouse mutants versus control—that is, hyperconnectivity). **b**, Top-view brain maps showing fMRI connectivity differences (with Cohen’s *d* > 0.5) in the 20 autism-related mouse models relative to wild-type littermates. Blue/light blue indicates hypoconnectivity; red/yellow indicates hyperconnectivity in mutants. **c**, Heatmap of Cohen’s *d* values across brain regions. Hierarchical clustering revealed two distinct connectivity subtypes: a hyperconnectivity cluster (*n* = 9 mouse models) and a hypoconnectivity cluster (*n* = 11 mouse models). **d**, Coronal brain views highlighting voxels with consistent hypoconnectivity or hyperconnectivity in the two fMRI dysconnectivity subtypes. Color intensity reflects the number of autism-related mouse models showing fMRI dysconnectivity (blue: hypoconnectivity, top panel; red/yellow: hyperconnectivity, bottom panel) in each subtype (threshold: Cohen’s *d* > 0.8). Amy, amygdala; BF, basal forebrain; Cing, anterior cingulate; CPu, caudoputamen; HPC, hippocampus; Hyper, hyperconnectivity; Hypo, hypoconnectivity; Hypt, hypothalamus; Ins, insula; MB, mid-brain; mPFC, medial prefrontal cortex; Motor ctx, motor cortex; NAc, nucleus accumbens.

Voxelwise quantification of fMRI dysconnectivity differences (that is, mutant versus wild-type) across the 20 autism models revealed a spectrum of dysconnectivity, ranging from marked hypoconnectivity (that is, decreased fMRI connectivity in mutants) to marked hyperconnectivity (that is, increased fMRI connectivity in mouse models) (Fig. 1a,b). This finding indicates that biological variability is a key determinant of connectivity heterogeneity in mouse models relevant to autism. Interestingly, although most models exhibited a combination of both hyperconnected and hypoconnected voxels, a clear polarization of the dysconnectivity landscape was also evident, with multiple mouse models exhibiting either predominant hypoconnectivity (for example, *En2*, *Shank3*, *22q11.2*, *16p11.2*, *Ube3A* and *Sgsh*) or predominant hyperconnectivity (for example, *Cdkl59* [ko], *Fmr1*, *Chd8*, *Tsc2* and *Il-6*) (Fig. 1a,b). In keeping with this notion, hierarchical clustering of fMRI dysconnectivity revealed two dominant subtypes characterized by robust hypoconnectivity (*n* = 11 mouse models) and hyperconnectivity (*n* = 9) (Fig. 1c), respectively. Given that our goal was to identify dominant brain dysconnectivity patterns, all our subsequent analyses focused on these two prominent clusters (Methods).

This two-cluster approach also provided sufficient mouse models per subtype to generate robust (Cohen’s *d* > 0.8) subtype-level dysconnectivity maps. These were obtained through a conjunction analysis of all fMRI dysconnectivity patterns, each corresponding to a specific mouse model, within each subtype (Fig. 1d). The resulting cross-etiological dysconnectivity maps represent brain regions that are most consistently vulnerable to hypoconnectivity or hyperconnectivity in each subtype.

**Extended Data Fig. 1:**
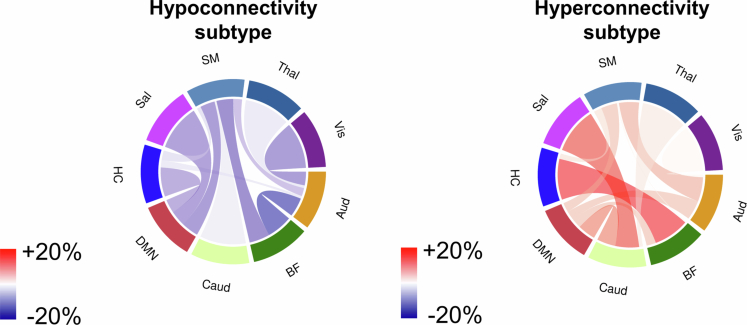
fMRI connectivity in 20 autism mouse models clusters into two dominant hypo- and hyperconnectivity subtypes. Chord diagrams showing atypical fMRI network structure in hypo- (left) and hyperconnectivity (right) subtypes, upon regression of mean fMRI connectivity across all parcellation units.

Interestingly, these subtype-specific maps revealed both overlapping and distinct regional patterns. Some anatomical regions, including the medial prefrontal cortex, striatum and basal forebrain, were susceptible to either hypoconnectivity or hyperconnectivity across subtypes. Other regions showed, instead, subtype-specific atypicalities. For example, the hippocampus and amygdala were predominantly implicated in the hyperconnectivity subtype, whereas the hypothalamus and somatomotor cortex were atypical in the hypoconnectivity subtype. Notably, network comparisons between mutant and control mice within each subtype revealed the engagement of dissociable large-scale network systems, indicating distinctly atypical patterns of functional organization (Extended Data Fig. 1). The hypoconnectivity subtype displayed widespread reductions in fMRI connectivity among salience, default mode and hippocampal networks, whereas the hyperconnectivity subtype showed predominant increases in fMRI coupling among limbic, salience and subcortical systems. These results support the notion that the two rodent autism-relevant subtypes encompass distinct network architectures, as supported also by their negligible spatial overlap (Pearson’s *r* = 0.23, *P* = 0.18). More broadly, these findings demonstrate that the dysconnectivity landscape across 20 autism mouse models segregates into two dominant, functionally opposed patterns.

### fMRI hypoconnectivity and hyperconnectivity reflect dissociable biological pathways

**Fig. 2:**
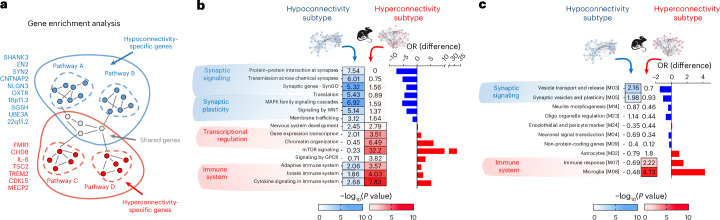
Distinct signaling pathways underlie fMRI connectivity subtypes in rodents. **a**, Illustrative schematic of gene enrichment analysis used to link autism-relevant pathways to rodent hypoconnectivity and hyperconnectivity subtypes. Autism risk genes and immune factors (that is, IL-6) modeled in mouse lines associated with either subtype (listed in blue and red typeface, respectively) were used as seed genes to generate mouse line-specific protein−protein interactomes. Within each subtype, these interactomes were then concatenated, and, upon removal of shared genes, we generated two subtype-specific gene interactomes. Filled circles represent individual genes; gray links indicate gene interactions; dashed lines delineate individual interactomes; and solid lines outline the two concatenated interactomes. **b**, Heatmap displaying significant enrichment for autism-relevant pathways in the two interactomes. **c**, Heatmap displaying significant enrichment for modules of genes differentially expressed in autism^16^ for each of the two interactomes. The ORs for hypoconnectivity subtype are shown in the left column (blue coloring); those for the hyperconnectivity subtype are shown in the right column (red coloring). Cell borders in **b** and **c** indicate that enrichment is significant at *q*_FDR_ < 0.05 (two-sided). Bar plots show the difference between the ORs of the hypoconnected and hyperconnected interactomes. Blue means predominant enrichment of the hypoconnected interactome; red means predominant enrichment of the hyperconnected interactome (right). We report the list of genes belonging to each interactome and the pathways we probed in Supplementary Table 2.

The identification of dominant hypoconnectivity and hyperconnectivity subtypes in our mouse database enabled us to empirically investigate whether different patterns of autism-relevant dysconnectivity reflect dissociable biological pathways. To test this hypothesis, we generated two aggregate sets of molecular pathways, each specifically associated with fMRI hypoconnectivity or hyperconnectivity. We began by constructing in silico two protein−protein mega-interactomes, each comprising the genetic and immune-related etiologies belonging to either the hypoconnectivity or the hyperconnectivity subtype, along with their interacting genes (Fig. 2a). We next filtered out the genes present in both mega-interactomes, retaining only those uniquely represented in each subtype-specific gene set. This step allowed us to focus our subsequent investigations on pathways that may specifically be associated with hypoconnectivity or hyperconnectivity subtypes. We finally applied a gene ontology analysis to identify, for each of the resulting interactomes, the prevalence of molecular pathways known to be dysregulated in autism^36^.

Using this approach, we identified a composite set of autism-relevant molecular pathways linked to hypoconnectivity or hyperconnectivity. Notably, these pathways were distinctly dissociable (Fig. 2b). Specifically, the hypoconnectivity subtype exhibited prominent enrichment for multiple synaptic-related ontologies, including genes involved in protein−protein interaction at the synapse (odds ratio (OR) = 7.54, *P*_FDR_ = 10^−9^), transmission across chemical synapses (OR = 6.01, *P*_FDR_ = 10^−20^) and synaptic functioning (SynGO, OR = 5.32, *P*_FDR_ = 10^−42^). This subtype was also significantly enriched for pleiotropic molecular effectors that play an essential role in the control of synaptic homeostasis and plasticity, such as MAPK signaling (OR = 6.92, *P*_FDR_ = 10^−43^), membrane trafficking (OR = 3.12, *P*_FDR_ = 10^−8^), protein translation (OR = 5.43, *P*_FDR_ = 10^−29^) and WNT signaling (OR = 5.14, *P*_FDR_ = 10^−19^)^37,38^. Immune-related mechanisms, such as innate (OR = 1.86, *P*_FDR_ = 10^−8^), adaptive (OR = 2.06, *P*_FDR_ = 10^−13^) and cytokine signaling (OR = 2.68, *P*_FDR_ = 10^−11^), were, instead, only weakly represented in this subtype.

**Extended Data Fig. 2:**
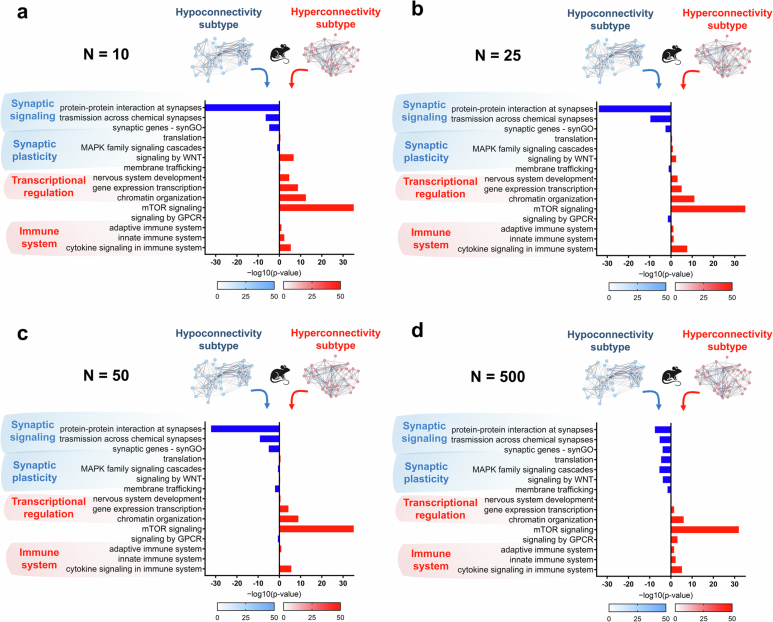
Sensitivity analysis of enrichments with varying size of PPI interactomes. Biological pathway analysis with PPI interactomes of increasing size, that is, **a**) n = 10, **b**) n = 25, **c**) n = 50 and **d**) n = 500. Data in fig. 2 reports results obtained with n = 100. Plots show differences in OR between enrichments of the hypoconnectivity and hyperconnectivity subtypes.

By contrast, no synaptic-specific mechanisms were enriched in the hyperconnectivity interactome (all synaptic ontologies, *q*_FDR_ > 0.05, except for the pleiotropic mTOR pathway, OR = 32.2, *P*_FDR_ = 10^−42^). This interactome, however, presented robust enrichment for immune-related pathways, such as cytokine signaling (OR = 7.83, *P*_FDR_ = 10^−39^), innate immune response (OR = 4.03, *P*_FDR_ = 10^−33^), adaptive immune response (OR = 3.57, *P*_FDR_ = 10^−14^), as well as transcriptional mechanisms (for example, chromatin organization, OR = 6.49, *P*_FDR_ = 10^−29^, and gene expression transcription, OR = 3.51, *P*_FDR_ = 10^−29^) (Fig. 2b). Notably, most of these enrichments appeared to be robust to interactome stringency, as we found them to be fully preserved after reducing the number of interactors from 100 to 50, 25 and 10 or by expanding the depth of STRING expansion up to 500 interacting genes (Extended Data Fig. 2). Not surprisingly, pleiotropic plasticity-related pathways (for example, MAPK, translation and WNT) became enriched only at moderate to high interactome depths (that is, ≥100 interactors), consistent with the need to include secondary interactors to capture convergence across these functionally diverse molecular networks.

To assess the robustness of these results against different sets of gene ontologies, we repeated enrichment analyses using gene modules previously described to be dysregulated in postmortem brains of individuals with autism^16^. Consistent with our original findings, we found that the hypoconnectivity subtype was robustly enriched for modules containing genes involved in regulation of synaptic activity, such as vesicle transport and release (OR = 2.16, *P*_FDR_ = 10^−8^) and synaptic vesicles and plasticity (OR = 1.98, *P*_FDR_ = 10^−4^). The hyperconnectivity interactome was, instead, significantly enriched for gene modules involved in immune signaling, such as immune response (OR = 2.2, *P*_FDR_ = 10^−3^) and microglia (OR = 4.73, *P*_FDR_ = 10^−17^) (Fig. 2c). As a note, because the Gandal et al. dataset^16^ did not include modules unequivocally related to transcriptional regulation, replicability of this pathway could not be evaluated. Taken together, these results indicate that dissociable mechanisms underscore diverging fMRI patterns of dysconnectivity in autism, with synaptic dysfunction being linked to fMRI hypoconnectivity and immune and transcriptional dysregulation being more prominently associated with fMRI hyperconnectivity.

### Reproducible hypoconnectivity and hyperconnectivity subtypes can be identified in human data

**Fig. 3:**
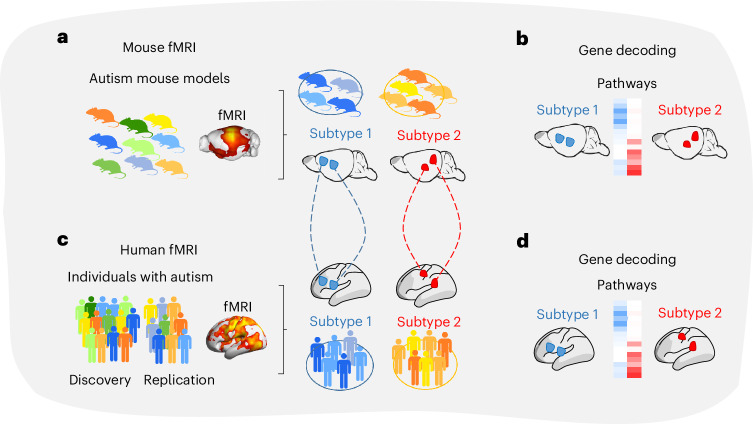
Cross-species identification of autism-related dysconnectivity subtypes. Schematic illustration of the workflow we used to identify hypoconnectivity and hyperconnectivity subtypes across species. **a**, We applied data-driven hierarchical clustering analyses to our rodent database and identified dominant hypoconnectivity and hyperconnectivity subtypes across autism mouse models relevant to autism. For each subtype, we generated a dysconnectivity prior mask, consisting of a set of anatomical regions exhibiting hypoconnectivity or hyperconnectivity across models. **b**, Gene enrichment analyses were used to uncover molecular pathways associated with hypoconnectivity versus hyperconnectivity in rodent autism models. **c**, In humans, we computed fMRI dysconnectivity by comparing resting-state fMRI data of individuals with autism versus neurotypical individuals. Leveraging the cross-species translatability of fMRI, we used a regionwise approach to quantify fMRI dysconnectivity in individuals with autism relative to neurotypical individuals. Specifically, we selected fMRI scans exhibiting hypoconnectivity or hyperconnectivity in human brain regions corresponding to the dysconnectivity priors identified in rodents (Supplementary Fig. 1). **d**, Finally, we performed gene enrichment analyses to investigate whether brain decoded genes from each map were enriched for molecular ontologies or gene modules known to be associated with autism.

As summarized in Fig. 3, after the identification of the two dominant brain connectivity subtypes in the rodent database, we probed the relevance of these findings in humans. Specifically, leveraging the cross-species translatability of fMRI connectivity^23,28^, we asked whether analogous hypoconnectivity and hyperconnectivity subtypes could be identified in a large database of fMRI scans acquired in individuals with idiopathic autism. This approach was followed by a gene decoding of the autism-related hypoconnectivity and hyperconnectivity maps obtained in the human data^28^.

We examined an aggregated dataset of human low-motion resting-state fMRI data, comprising *n* = 940 individuals on the autism spectrum (age range, 5–30 years) and *n* = 1,036 age-matched neurotypical controls. The probed datasets comprised 38 data collections, 37 of them selected from the Autism Brain Imaging Data Exchange (ABIDE) repositories and one being a newly collected sample aggregated at the Child Mind Institute (CMI)^39–42^ (Table 2). To evaluate subtype reproducibility, we a priori split this aggregated dataset into a discovery dataset (78.5% of the aggregate sample, *n* = 744 individuals with autism and *n* = 807 neurotypical controls) and a replication dataset (21.5% of the aggregate dataset, *n* = 196 individuals with autism and *n* = 229 neurotypical controls) (Methods) matched for diagnosis, sex, age and in-scanner head motion.

**Table 2:** Breakdown of demographics and clinical scores by diagnostic group: autism and neurotypical

	Autism	Neurotypical	Group comparisons, statistics and *P* values
Sample size, *n*	940	1,036	
Data collections^a^, *n*	38	35	
Sex, M, F, *n*	811, 129	774, 262	*χ*_1_^2^ = 41.5, *P* < 0.001
Age, years	13.7 (5.2) [5.1−29.2]	14.1 (5.5) [5.9−29.9]	*t*_1,974_ = 1.93, *P* = 0.06
Full-scale IQ^b^, standard score	105.9 (16.9) [41−149]	113.1 (12.8) [71−151]	*t*_1,785_ = 10.3, *P* < 0.001
Verbal IQ^c^, standard score	105.7 (17.8) [42−180]	113.8 (13.7) [67−156]	*t*_1,516_ = 9.9, *P* < 0.001
Non-verbal IQ^d^, standard score	105.1 (17.3) [37−157]	109.5 (13.7) [62−155]	*t*_1,581_ = 5.70, *P* < 0.001
ADOS SA^e^, CSS	6.8 (2.1) [1–10]	–	–
ADOS RRB^f^, CSS	7.1 (2.5) [1–10]	–	–
ADOS total^g^, CSS	6.8 (2.2) [1–10]	–	–
Median FD, mm	0.067 (0.04) [0.01−0.19]	0.062 (0.03) [0.01−0.19]	*t*_1,974_ = 2.97, *P* = 0.003
Psychiatric co-occurrences in autism^h^, *n* (%)	241 (55.3%)	–	–
Psychoactive medication use^i^, *n* (%)	246 (32%)	5 (0.6%)	*χ*_1_^2^ = 291.0, *P* < 0.0001

For continuous variables, group mean and standard deviations are reported in parentheses, and minima and maxima are reported in brackets.

**Fig. 4:**
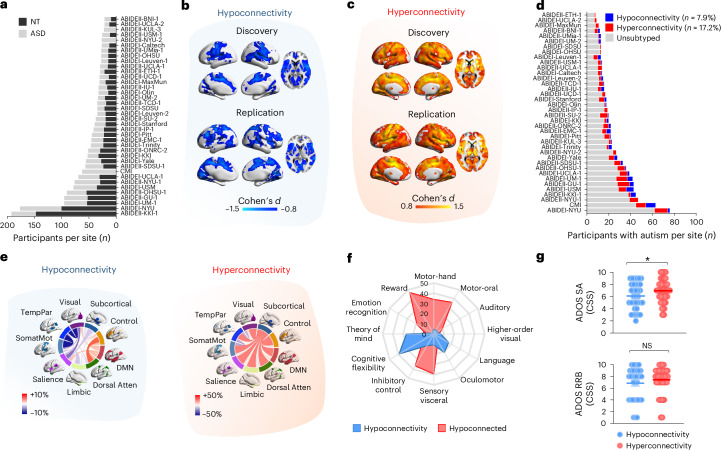
Replicable hypoconnectivity and hyperconnectivity subtypes can be identified in autism. **a**, Sample size distribution by data collection (ASD; light gray, NT; dark gray); ASD total *n* = 940; NT total *n* = 1,036; *n* = 38 data collections across 23 sites (Table 2). **b**, Hypoconnectivity subtype maps in discovery (top subpanel: *n* = 55, 7.4%) and replication (bottom subpanel: *n* = 19, 9.7%) datasets. Blue indicates regions exhibiting fMRI hypoconnectivity compared to NTs (Cohen’s *d* ≤ 0.8). **c**, Hyperconnectivity subtype maps in discovery (top subpanel: *n* = 124, 16.7%) and replication (bottom subpanel: *n* = 38, 19.4%) datasets. Red/yellow indicates fMRI hyperconnectivity (Cohen’s *d* ≥ 0.8). **d**, Distribution of individuals with autism included in the hypoconnectivity or hyperconnectivity subtypes in the aggregated (discovery plus replication) autism dataset (blue: hypoconnectivity subtype, *n* = 74 across *n* = 28 data collections; red: hyperconnectivity, *n* = 162 across *n* = 38 data collections), as well as those not assigned to either subtype (light gray *n* = 704 across all 38 collections). **e**, Connectograms showing atypical fMRI network structure in hypoconnectivity (left) and hyperconnectivity (right) subtypes (upon regression of mean fMRI connectivity across 414 parcellation units)^44,87^. Link thickness is proportional to the number of between-network edges displaying a significant difference in ASD versus NT groups (red: increased connectivity, blue: decreased connectivity; *t* > 3.1, NBS corrected^88^ at *P* < 0.05, two-sided). **f**, Radar plot showing the percentage of overlap (range, 0–50%) between subtype mean regressed connectivity difference maps and 12 neurocognitive ontology probability maps. **g**, Autism subdomain severity scores (top subpanel: SA; bottom subpanel: RRB) based on ADOS (Methods) for each subtype. SA: hypoconnectivity subtype *n* = 33, mean=6.1 ± 2.2; hyperconnectivity subtype, *n* = 84, mean = 7.0 ± 1.8; *t*_115_ = 2.37, *P*_uncorr_ = 0.019 two-sided, *P*_FDR_ = 0.030. RRB: hypoconnectivity subtype *n* = 34, mean = 6.9 ± 2.8; hyperconnectivity subtype, *n* = 85, mean = 7.5 ± 2.2; *t*_117_ = 1.20, *P*_uncorr_ = 0.23 two-sided, *P*_FDR_ = 0.23. Error bars: s.e.m.; *P < 0.05. DMN, default mode network; Dorsal Atten, dorsal attention network; Limbic, limbic network; NS, not significant; NT, neurotypical; SA, social affect; RRB, restricted repetitive behaviors; Salience, salience network; SomatMot, somatomotor network; TempPar, temporoparietal network; Visual, visual network.

^a^The data aggregate included 38 data collections: 18 from ABIDE I (Caltech, KKI, Leuven-1, Leuven-2, MaxMun, NYU, OHSU, Olin, Pitt, SDSU, Stanford, Trinity, UCLA-1, UCLA-2, UM-1, UM-2, USM and Yale), 19 from ABIDE II (BNI-1, EMC-1, ETH-1, GU-1, IP-1, IU-1, KKI-1, KUL-3, NYU-1, NYU-2, OHSU-1, ONRC-2, SDSU-1, SU-2, TDC-1, UCD-1, UCLA-1, U-MIA-1 nd USM-1) and one from CMI; of these, three included data only from individuals on the autism spectrum (Fig. 4a).

^b^Full-scale IQ was available for *n* = 819 individual data of the autism diagnostic group and for *n* = 968 of the neurotypical group.

^c^Verbal IQ was available for *n* = 709 individual data of the autism diagnostic group and for *n* = 809 of the neurotypical group.

^d^Non-verbal IQ was available for *n* = 726 individual data of the autism group and for *n* = 857 of the neurotypical group.

^e^ADOS SA CSSs were available for *n* = 520 individuals in the autism group.

^f^ADOS RRB scores were available for *n* = 525 individuals in the autism group.

^g^ADOS total CSSs were available for *n* = 549 in the autism group (Methods).

^h^Number and percentage of individuals with autism with a diagnostic label of one or more psychiatric co-occurring condition. Psychiatric co-occurrences were assessed in a subset of data with an ASD diagnostic label across 13 collections (*n* = 436 individuals).

^i^Number and percentage of individuals taking psychoactive medications. Information on psychoactive medications was available for a subset of data (autism *n* = 777 and neurotypical *n* = 822), across 27 collections.

FD, framewise displacement; SA, social affect; RRB, restricted repetitive behaviors; *t*, unpaired *t*-test statistics; *χ*^2^, chi-square statistics.

To enable a cross-species extrapolation of our mouse results, we used a regional decoding approach focusing on evolutionarily conserved brain regions identified in the conjunction dysconnectivity maps of corresponding hypoconnectivity and hyperconnectivity rodent subtypes (Methods and Supplementary Fig. 1). This enabled us to identify two subgroups (that is, subtypes) of individuals with autism (Fig. 4a−d), each showing robust (Cohen’s *d* > 0.8) differences in fMRI connectivity relative to neurotypical controls, consistent with hypoconnectivity and hyperconnectivity subtypes observed in the rodent dataset. Together, the two subtypes accounted for 24.1% of the discovery autism dataset (*n* = 55 (7.4%) and *n* = 124 (16.7%) for the hypoconnectivity and hyperconnectivity subtypes, respectively). Quantifications of fMRI dysconnectivity confirmed that the evolutionary conserved regions showing significant hypoconnectivity or hyperconnectivity in humans were consistent with those we identified in corresponding rodent subtypes, thus validating our cross-species translation (Supplementary Fig. 2). To probe the replicability of these findings, we repeated our cross-species decoding on a replication dataset identified a priori. Analyses of this dataset revealed two hypoconnectivity and hyperconnectivity subtypes, accounting for 29.1% of the autism replication data (*n* = 19 (9.7%) and *n* = 38 (19.4%) for the hypoconnectivity and hyperconnectivity subtypes, respectively; Fig. 4b,c). The topography and connectivity profiles of the two subtypes were highly reproducible across the discovery and replication datasets (hypoconnectivity subtype, Dice coefficient = 0.74, *r* = 0.67; hyperconnectivity subtype, Dice coefficient = 0.96, *r* = 0.7) (Supplementary Fig. 3). Collectively, the identified subtypes accounted for 25.1% of the aggregated autism data examined (hypoconnectivity, *n* = 74 (7.9%) and hyperconnectivity, *n* = 162 (17.2%), upon aggregation of discovery and replication autism datasets—that is, *n* = 940). Across the combined sample, scans representing the hyperconnectivity subtype were present in all the data collections, whereas scans exhibiting hypoconnectivity were present in all but 10 data collections (Fig. 4d). To further corroborate the generalizability of these results, we repeated our cross-species decoding upon removal of the five largest collections of data of individuals with autism. Results were largely consistent with our primary findings (Methods and Supplementary Fig. 4); this suggests that our subtyping was not biased by the largest data collections.

### Hypoconnectivity and hyperconnectivity subtypes exhibit different network structure and are behaviorally dissociable

We further characterized the human subtypes in regard to their network structure, associated cognitive ontology maps^43^ and autism symptomatology. To increase statistical power, analyses were carried out on the aggregate dataset combining the subtyped discovery and replication cohorts. Network comparisons of autism versus neurotypical data within subtypes (Methods) revealed specific network atypicalities. Markedly increased subcortico−cortical connectivity (*t* > 3.1, *P* < 0.05; Fig. 4e) and reduced cortico−cortical connectivity among temporoparietal, visual and somatomotor networks were noted in the hyperconnectivity subtype. In this subtype, the largest differences included increased connectivity within subcortical regions (53% of node-to-node links) and their connections to the salience network (44% of the 414 cortical and subcortical parcellation units^44,45^). Conversely, the hypoconnectivity subtype displayed significant network-level differences (left panel, *t* > 3.1, *P* < 0.05) involving decreased connectivity between the somatomotor and temporoparietal networks (5% of links). Supporting a distinct network organization for the two subtypes, their connectivity matrices showed negligible spatial overlap (*r* = 0.07, *P* = 0.53). Interestingly, despite a few species-specific differences, these network patterns were broadly consistent with those observed in rodents, displaying similar directionality and organization of hypoconnectivity and hyperconnectivity across major systems. These findings indicate that the identified hypoconnectivity and hyperconnectivity patterns represent two functionally distinct subtypes characterized by different underlying brain network architecture. Consistent with this notion, reverse inference mapping^43^ revealed that the network structure of these two subtypes is associated with distinct ontology cognitive maps. Specifically, the connectivity map of the hypoconnectivity subtype overlapped with oculomotor, language and cognitive areas, whereas the hyperconnectivity map was associated with sensory, visceral, motor, reward and inhibitory control areas (Fig. 4f).

Finally, to assess the behavioral profile of the two autism-related subtypes, we compared autism symptom severity using Autism Diagnostic Observation Schedule, Second Edition 2 (ADOS-2)-based severity total and subtotal scores available in subsets of individuals (‘social affect’, *n* = 117; restricted and repetitive behavior (RRB), *n* = 119; total calibrated severity score (CSS), *n* = 125; Methods). On average, individuals in the hyperconnectivity subtype exhibited moderately increased total CSSs relative to individuals in the hypoconnectivity subtype (hypoconnectivity subtype *n* = 38, mean = 6.1 ± 2.5; hyperconnectivity subtype, *n* = 87, mean = 7.1 ± 1.9; *t*_123_ = 2.20, *P*_uncorr_ = 0.020, *P*_FDR_ = 0.030). As shown in Fig. 4g, comparisons of symptom subdomain severity scores revealed subtype differences for social affect but not for RRB scores. Exploratory analyses revealed no statistically significant differences between subtypes with respect to other available phenotypic data, including age, IQ, sex, psychiatric co-occurrence rates and medication status (Supplementary Table 1). Notably, fMRI connectivity strength correlated with ADOS scores (Supplementary Fig. 5). Taken together, these findings indicate that the identified hypoconnectivity and hyperconnectivity subtypes exhibit distinct functional network patterns and autism symptom severity.

### Hypoconnectivity and hyperconnectivity in humans recapitulate molecular pathways identified in rodent models

Given that similar connectivity subtypes exist in individuals with autism, we investigated whether they also reflect analogous molecular mechanisms. Using spatial gene decoding^46^, we identified two sets of subtype-specific genes that are spatially enriched in the identified hypoconnectivity and hyperconnectivity subtypes. We next filtered out the genes spatially enriched for both subtypes, retaining only those uniquely represented in each subtype-specific gene set. This step allowed us to focus our subsequent investigations on pathways more likely unique to either the hypoconnectivity or the hyperconnectivity subtype. We next asked whether these gene sets were enriched for autism-relevant transcripts and if they recapitulated the molecular pathways observed in mouse hypoconnectivity and hyperconnectivity subtypes.

**Fig. 5:**
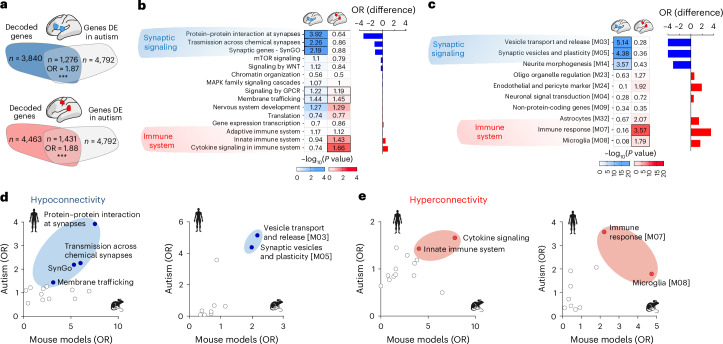
Hypoconnectivity and hyperconnectivity subtypes recapitulate synaptic and immune pathways modeled in mice. **a**, Venn diagrams showing enrichment between brain decoded genes (that is, genes spatially correlated with atypical connectivity patterns) and differentially expressed genes in autism^16^ for both subtypes. Blue/red areas report the number (*n*) of brain decoded genes for each subtype. Gray: differentially expressed genes; overlap (that is, enriched genes) is reported with corresponding ORs. ***P_FDR_ < 0.001. Heatmap of enrichments between brain decoded genes and autism dysregulated pathways (**b**) or modules of genes differentially expressed in autism (**c**)^16^. Left columns: hypoconnectivity (blue); right: hyperconnectivity (red). Color intensity indicates enrichment significance (−log *P* value). Thick borders mark significant enrichments (*q*_FDR_ < 0.05, two-sided). Bar plots show the difference between the ORs of the hypoconnected and hyperconnected interactomes. Blue means predominant enrichment of the hypoconnected interactome; red means predominant enrichment of the hyperconnected interactome (right). **d**, Left, scatter plot representation of OR of the molecular ontologies associated with mouse hypoconnectivity (*x* axis) versus OR of the same molecular ontologies decoded in autism hypoconnectivity map (*y* axis). Right, the same plot is also reported for ORs of genes differentially expressed in autism^16^. **e**, Left, scatter plot representation of OR of the molecular ontologies of mouse hyperconnectivity (*x* axis) versus OR of the same molecular ontologies decoded in autism hyperconnectivity map (*y* axis). Right, the same plot is also reported for ORs of genes differentially expressed in autism^16^. Pathways significantly enriched at *q*_FDR_ < 0.05 in both mouse models and autism are highlighted with blue (hypoconnectivity) or red (hyperconnectivity) shading. We report the list of brain decoded genes and the pathways that we probed in Supplementary Table 3. SFARI scores were derived from https://gene.sfari.org/. DE, differentially expressed; SFARI, Simons Foundation Autism Research Initiative.

Supporting the validity of our cross-species approach, both dysconnectivity subtypes showed significant spatial enrichment for genes differentially expressed in the postmortem cortex of individuals with autism (hypoconnectivity, OR = 1.87, *P*_FDR_ = 10^−3^; hyperconnectivity, OR = 1.88, *P*_FDR_ = 10^−3^; Fig. 5a (ref. ^16^)). Notably, neither subtype showed significant enrichment for genes associated with bipolar disorder, psoriasis, dementia, attention-deficit/hyperactivity disorder or schizophrenia (Supplementary Fig. 6).

Having established that the decoded gene sets were enriched for autism-associated genes, we next investigated their biological functions using pathway-specific gene ontology analysis. This analysis revealed that the two subtypes are associated with distinct biological pathways, mirroring specific molecular dysfunctions observed in the corresponding rodent connectivity subtypes (Fig. 5b,c). Specifically, brain decoded genes for the hypoconnectivity subtype showed robust enrichment for multiple synaptic ontologies, such as protein−protein interaction at the synapsis (OR = 0.92, *P*_FDR_ = 10^−6^), transmission across chemical synapsis (OR = 2.26, *P*_FDR_ = 10^−7^), SynGO (OR = 2.19, *P*_FDR_ = 10^−14^) and membrane trafficking (OR = 1.44, *P*_FDR_ = 10^−2^). By contrast, the hyperconnectivity subtype was specifically enriched for immune-related pathways, such as cytokine signaling (OR = 1.66, *P*_FDR_ = 10^−3^) and innate immune system function (OR = 1.43, *P*_FDR_ = 10^−2^) (Fig. 5b) but did not show enrichment for synaptic ontologies (all *P*_FDR_ > 0.05). Unlike what was observed in our rodent dataset, no enrichment for transcriptional mechanisms was observed in this connectivity subtype. A replication of gene enrichment analysis using gene modules differentially expressed in the autistic brain^16^ revealed significant synaptic enrichment in the hypoconnectivity subtype (for example, vesicle transport and release, OR = 5.14, *P*_FDR_ = 10^−56^; synaptic vesicles and plasticity, OR = 4.38, *P*_FDR_ = 10^−26^) and immune-related enrichment in the hyperconnectivity subtype (for example, immune response, OR = 3.57, *P*_FDR_ = 10^−13^; reactive microglia, OR = 1.79, *P*_FDR_ = 10^−2^) (Fig. 5c). These results suggest cross-species conservation of the pathological pathways associated with the fMRI dysconnectivity subtypes. Similarly, we also found a broad correspondence between the ontologies or gene modules most robustly enriched in rodent dysconnectivity subtypes and those more prominently enriched in the corresponding human subtypes (Fig. 5d,e). Collectively, these results corroborate the mechanistic validity of our cross-species decoding and shed light on the molecular dysfunctions underlying two prominent and reproducible brain dysconnectivity subtypes in autism.

## Discussion

Using cross-species functional neuroimaging in large cohorts, we biologically decoded heterogeneous fMRI dysconnectivity patterns related to autism into two reproducible subtypes: one predominantly characterized by whole-brain hypoconnectivity, the other by widespread hyperconnectivity. Notably, gene decoding and enrichment revealed that the identified hypoconnectivity and hyperconnectivity subtypes are associated with synaptic dysfunction and immune-related mechanisms, respectively. From a translational standpoint, our results demonstrate the feasibility of using cross-species approaches to empirically decode a widely investigated neuroimaging phenotype in autism into mechanistically dissociable subtypes. Our findings also support the notion that heterogeneous brain dysconnectivity in autism reflects its underlying etiological heterogeneity, thereby providing empirical support for ongoing efforts in neurobiological subtyping of the spectrum^18,20,22^.

Building upon and expanding our previous rodent fMRI database^34^, we investigated a broad range of etiologically relevant autism mouse models. Our findings provide compelling evidence that atypical functional connectivity, along with its cross-etiological heterogeneity, is a defining pathophysiological hallmark of autism and is associated with distinct signaling pathways. Leveraging translationally relevant aggregate measures of dysconnectivity, our cross-species analyses uncovered novel biological insights into the molecular underpinnings of autism-relevant fMRI dysconnectivity. The enrichment in multiple synaptic ontologies found in the hypoconnectivity subtype implicates a central role of synaptic dysfunction in altering large-scale fMRI connectivity. This phenomenon was recently investigated at both theoretical and experimental levels, revealing a putative direct covarying relationship between excitatory synaptic density and aggregative fMRI connectivity measures like those used here^26,28^. The observation of decreased excitatory spine density in multiple mouse lines within the hypoconnectivity subtype, such as *Shank3* (ref. ^47^), *Cntnap2* (ref. ^48^), *Syn2* (ref. ^49^), 16p11.2 (ref. ^27^) and 22q11.2 (ref. ^28^), broadly supports this hypothesis and suggests that alterations in synaptic homeostasis (putatively leading to reduced synaptic density) may represent a plausible neurocellular marker for the observed hypoconnectivity.

The robust cross-species association between hyperconnectivity and immune-related signaling is also of great interest, as it suggests that various immune-mediated pathways converge to drive atypical functional coupling in the mammalian brain. This may occur through immune-mediated alterations of excitatory or inhibitory function^50,51^, microstructural white matter abnormalities^52^, microglial-induced alterations in axonal wiring and synaptic pruning^53^ as well as immune-related disruption of synaptogenesis^54^. This framework provides a testable mechanistic account for prior human fMRI evidence of hyperconnectivity linked to vulnerabilities in social reciprocity in individuals with autism^55^, supporting the translational relevance of our findings.

Importantly, multiple immune-related mechanisms have been shown to directly affect synaptic maturation and homeostasis^56^. Within this framework, fMRI hyperconnectivity may thus partly reflect an immune-related excess of excitatory synapses. The observation that multiple mouse models within the hyperconnectivity subtype, such as *Cdkl5* (ref. ^57^), *Tsc2* (ref. ^58^) and *Chd8* haploinsufficient mice^59^, as well as immune-related models such as mice prenatally treated with IL-6 (ref. ^60^) and *Trem2*-deficient mice^61^, have been reported to exhibit increased synaptic density—at least at early postnatal stages—aligns with this notion and corroborates emerging evidence linking synaptic dysfunction to macroscale fMRI dysconnectivity^26,28^.

Neuromanipulation studies in rodents^24^ also enabled us to speculate on the potential neurophysiological determinants of the observed fMRI dysconnectivity. Specifically, using chemogenetics and multielectrode electrophysiological recordings, we recently described an inverse relationship between cortical excitability and fMRI connectivity. We found that increased neural firing and cortical excitability may counterintuitively lead to reduced fMRI connectivity^62^, whereas decreased cortical excitability can lead to increased fMRI connectivity^63^. According to this model, fMRI hypoconnectivity and hyperconnectivity subtypes may thus reflect broadly increased cortical excitability or excessive inhibition, respectively. In this respect, our results provide a systems-level framework that aligns with, and extends, the longstanding theory of excitation–inhibition (E/I) imbalance in autism^64,65^. By linking molecular and transcriptomic signatures to opposing patterns of fMRI network coupling, our findings suggest that E/I imbalance may manifest at the macroscale as divergent modes of network dysconnectivity. The possible coexistence of contrasting excitatory dysfunction within the autism spectrum would be consistent with the recent identification of electrophysiologically opposed autism subtypes characterized by increased and decreased excitability as measured with electroencephalography (EEG)^66^. This convergence across modalities suggests that E/I imbalance in autism may not be a unitary phenomenon but, rather, a multidimensional construct whose expression can vary across individuals and developmental stages, potentially explaining the heterogeneity of autism-related brain connectivity. This hypothesis warrants further empirical testing in both rodents and humans and could have important implications for autism stratification or therapy.

In addition, we note that large-scale fMRI connectivity can also be modulated by neurochemical and neuromodulatory systems. Differences in cholinergic, serotonergic or dopaminergic tone, originating from basal forebrain, thalamic or hypothalamic nuclei that appear to be affected in both subtypes (Fig. 1d), may, therefore, contribute, directly or indirectly, to the observed patterns of hypoconnectivity and hyperconnectivity, either by shaping cortical excitability or through vascular mechanisms influencing blood-oxygen-level-dependent (BOLD) fMRI signal^67–69^. Future studies combining fMRI with direct measures of neuromodulatory activity will be essential to clarify the contribution of these systems to large-scale connectivity alterations in autism.

Interestingly, our functional partitioning does not appear to directly or unequivocally reflect underlying anatomical changes in mice. In a recent large-scale anatomical clustering study encompassing 135 mouse models, including 10 examined here^70^, hypoconnected and hyperconnected models were found to be evenly distributed across three (out of four) distinct neuroanatomical subgroups, with no preferential enrichment for either connectivity profile. This dissociation indicates that functional and structural alterations capture partly independent components of the autism phenotype, reinforcing the need for multidimensional frameworks that integrate fMRI connectivity with anatomical and molecular readouts.

Although our cross-species approach primarily focused on two major hypoconnectivity and hyperconnectivity subtypes, the relatively coarse partitioning that we implemented may have hindered the detection of additional dysconnectivity subtypes and more nuanced sets of molecular alterations. For example, one set of pathways that we were unable to reliably decode in humans is transcriptional dysregulation, which, in mice, co-clustered with immune dysfunction. It is conceivable that the patterns produced by these two distinct pathways could become further dissociable by extending our database to include a larger number of mouse models. Alternatively, transcriptional dysregulation may not be reliably linkable to distinctive pattern of dysconnectivity, owing to the highly stochastic nature of its developmental outcomes^71^. This hypothesis would be consistent with the phenotypic variability observed upon deletion of the chromatin regulator *Chd8* on different genetic backgrounds^72^. In keeping with this view, the lack of a robust chromatin/transcriptional signal in our gene decoding of human fMRI maps suggests that such mechanisms are unlikely to be dominant contributors to large-scale dysconnectivity in idiopathic autism. It is also important to note that our enrichment results should not be interpreted as indicating absolute exclusivity of molecular pathways to either subtype. Rather, they suggest that the synaptic and immune/transcriptional mechanisms we identified may shape the polarity of connectivity alterations toward hypoconnectivity or hyperconnectivity, on top of a heterogeneous biological landscape in which multiple mechanisms may coexist. This nuance is consistent with the complex etiological landscape of autism and underscores the value of a comparative, rather than a categorical, interpretation of our results.

Notwithstanding the large evolutionary distance between rodents and humans, and the current inability to effectively model common polygenic variants in rodents, our cross-species approach allowed us to successfully decode approximately one-fourth of the fMRI scans acquired in individuals with non-syndromic, idiopathic autism (for whom no single genetic mutation was known). This finding underscores the translational potential of our approach and suggests that prevalent autism-relevant pathophysiological alterations can be decoded in human fMRI scans despite key cross-species differences. This result also reinforces previous evidence of substantial overlap between pathways dysregulated in idiopathic autism (which are thought to reflect the cumulative contribution of common genetic variants^73^) and those affected by the rare, high-penetrance mutations that can be reliably modeled in rodents^16,30^.

Although our current framework does not yet capture the full spectrum of autism-related brain dysconnectivity, our results provide a solid foundation for expanding this approach. The integration of functional neuroimaging in genetically characterized individuals with autism, alongside future expansions of our rodent database to encompass additional mutations and mechanisms, will be crucial for further refining these insights, with the potential of revealing additional dysconnectivity subtypes beyond the dominant hyperconnectivity and hypoconnectivity subtypes that we described here. Future studies employing sex-balanced cohorts will also be essential to systematically assess potential sex-by-subtype interactions and to determine whether similar biological principles generalize across sexes. We also note that our findings do not imply that scans lacking subtype classification necessarily exhibit typical connectivity, nor do they suggest that putative connectivity changes in this population would be unrelated to underlying etiology. Rather, they suggest that atypical fMRI connectivity in autism exists along a more subtle continuum, often manifesting in ways that are not readily detectable via conventional case−control comparisons. By refining our analytical approaches and expanding our datasets, future studies may capture a broader range of atypical connectivity subtypes, further enriching understanding of the etiological diversity of autism. We finally note that the primary goal of our study is not immediate clinical application but, rather, to establish a conceptual link between macroscale fMRI connectivity patterns and underlying biological variability. At present, the two subtypes should be regarded as observational; nevertheless, they provide a framework for future work to link connectivity-defined subtypes with specific biological or neurophysiological alterations and, ultimately, to stratify endophenotypes in mechanism-targeted studies. Substantial additional validation will be required before such approaches can inform clinical translation.

The observation of symptom severity differences between the two autism subtypes in the face of their largely opposing functional architecture supports future studies aimed at examining more fine-grained symptom scores. Indeed, deeper and more harmonized phenotyping are critical steps for future translational and clinical subtype validation. Nevertheless, our demonstration that fMRI can encode for complex biological pathways suggests that there is broad scope for fMRI to be used in neurological and psychiatric research, beyond the mere establishment of brain−behavior associations.

In conclusion, our results redefine autism as a neurodevelopmental condition characterized by dissociable neurobiological subtypes and provide empirical evidence linking the phenotypic heterogeneity of the autism spectrum to its underlying biological variability. Our work also offers novel insights into the biology of autism, highlighting a role of synaptic and immune-related mechanisms in driving autism-related functional dysconnectivity. Finally, from a methodological standpoint, our cross-species approach provides an advanced translational framework for a multidimensional, biologically grounded stratification of autism. Our database is openly available to the research community, supporting future investigations into autism-related connectivity alterations.

## Methods

### Statistical considerations

No statistical methods were used to predetermine sample sizes. Sample sizes were determined by the availability of data in the aggregated mouse and human fMRI collections analyzed here and are similar to (or exceed) those used in prior large-scale neuroimaging studies. Most data distribution was assumed to be normal, but this was not formally tested. Analyses focused primarily on effect sizes (Cohen’s *d*), which can be computed without strict distributional assumptions; where appropriate, individual data points are shown. Randomization was not applicable to group assignment, as experimental groups were defined by genotype (mouse datasets) or diagnosis (human datasets), and data processing and analyses were applied identically across groups. Data analysis was performed using automated, scripted pipelines applied identically to all datasets; group labels were coded during processing and decoded only after the primary analyses were completed.

### Mouse studies

#### Ethics statement

All study procedures were approved by the institutional review board and are in accordance with the ethical standards of the Declaration of Helsinki of 1975, as revised in 2008. All experiments performed at Istituto Italiano di Tecnologia Rovereto (IIT) were conducted following Italian law (DL 27/1992 and DL 26/2014, EU 63/2010, Ministero della Sanità, Roma) and the recommendations in the Guide for the Care and Use of Laboratory Animals of the National Institutes of Health. All experiments performed at ETH Zürich (ETH) were in accordance with Swiss federal guidelines for using animals in research and under licensing from the Zürich Cantonal Veterinary Office. Animal research protocols were also reviewed and approved by the animal care committees at IIT (University of Trento) and ETH. At both sites, mice were group housed under controlled temperature (21 ± 1 °C) and humidity (60 ± 10%) and maintained on a standard 12-hour light/dark cycle. Food and water were provided ad libitum.

#### Autism mouse models

As detailed in Table 1, our collection of fMRI scans in 20 autism-relevant models includes retrospectively aggregated data from published and unpublished experiments across two laboratories. Specifically, 12 mouse models were scanned at IIT Rovereto and eight at ETH Zürich. Each study included mice with autism-relevant alterations and wild-type control littermates. Seventeen of these models carried genetic alterations associated with autism. The remaining included a model of environmental autism risk factor to maternal exposure to IL-6 (ref. ^60^); a model for TREM2 deficiency characterized by microglial defects and autistic-like behavioral phenotype^61^; and inbred BTBR mice^89^. The BTBR mouse line is characterized by congenital agenesis of the corpus callosum, a neuroanatomical trait associated with high prevalence in autism. A cohort of age-matched C57B6J mice was imaged during the same fMRI session of the BTBR mice and represents the reference control for this line^90^.

#### Resting-state fMRI

Mice were scanned on a BioSpec 70/16 small animal magnetic resonance system (Bruker BioSpin MRI) using controlled sedation^91,92^. Scans from IIT Rovereto were obtained with a 72-mm birdcage transmit coil and a custom-built saddle-shaped four-element coil for signal reception. Scans from ETH Zürich were obtained with a cryogenic quadrature surface coil (Bruker BioSpin AG). Standard adjustments included calibration of the reference frequency power and the shimming using MapShim (Paravision). Resting-state fMRI BOLD timeseries were acquired using a standard echo planar imaging sequence, as detailed previously^34^. In all fMRI acquisitions, controlled sedation was obtained using either isoflurane (0.5%) + medetomidine (0.05 mg kg^−1^) for data acquired at ETH Zürich^93^ or halothane (0.75%) for data acquired at IIT Rovereto^91,94^, with the exception of *Oxtr*-knockout, which was imaged under isoflurane and medetomidine.

### Resting-state fMRI connectivity mapping

Before mapping functional connectivity, we preprocessed the fMRI timeseries of the autism-related mouse models and wild-type control littermates. Preprocessing encompassed two sequential steps: core preprocessing and denoising. Core preprocessing included the following steps (with the software and function employed in parentheses). The initial 50 volumes of the timeseries were removed to allow for T1 and gradient thermal equilibration effects (AFNI 3dTcat). BOLD timeseries were then despiked (AFNI 3dDespike), motion corrected (FSL mcflirt), skull stripped (FSL bet) and spatially normalized with affine and diffeomorphic registration (ANTS antsRegistration + ANTS antsApplyTransforms) to a skull-stripped reference BOLD template. The denoising pipeline included the following steps: motion traces of six head realignment parameters (three translations + three rotations) and mean ventricular signal (corresponding to the averaged BOLD signal within a reference ventricular mask; FSL fslmeants) were used as nuisance covariates and regressed out from each timecourse (FSL fsl_regfilt). Then, nuisance regressed timeseries underwent band-pass filtering to a frequency window of 0.01−0.1 Hz (AFNI 3dBandpass) and spatial smoothing with a full width at half maximum (FWHM) of 0.6 mm (AFNI 3dBlurInMask).

fMRI connectivity was quantified on preprocessed timeseries using a voxelwise computation of weighted degree centrality for all mice^35^. We refer to this parameter here as ‘global connectivity’^28,95^. Global connectivity corresponds to the mean temporal Pearson’s correlation between a given voxel and all the other voxels within the brain^96^. This approach allowed us to obtain spatially unbiased connectivity mapping without the constraints of preimposed anatomical boundaries. Global connectivity is also amenable to direct cross-species translation^26,27^. Pearson’s correlation scores were first transformed to *z*-scores using Fisher’s *r*-to-*z* transform and then averaged across voxels to yield final global connectivity strength. Finally, differences in global connectivity strength between each autism model and its control littermates were quantified at the voxel level using Cohen’s *d*. Cohen’s *d* global connectivity maps for each autism-relevant mouse model were then plotted using global histogram analysis as implemented in the R package ‘ggplot’. Voxelwise global connectivity mapping was implemented using custom code in Python 3.

### fMRI dysconnectivity subtyping in autism-relevant mouse models

To identify cross-etiological fMRI dysconnectivity patterns, we applied cluster analysis to our Cohen’s *d* global connectivity maps. To this end, after vectorizing those statistical maps, we created a connectivity matrix, where rows are autism-relevant mouse models and columns are brain voxels. We then applied agglomerative hierarchical clustering to identify mouse models exhibiting similar Cohen’s *d* global connectivity maps, as implemented in the R package ‘heatmap.2’. A dendrogram was used to visualize the degree of similarity between autism-relevant mouse models and to demarcate clusters. Similarity was quantified by using Euclidean distance. To visualize the homogeneity of mouse fMRI dysconnectivity for a varying number of clusters, we plotted the within-group sum of square (WGSS) of fMRI dysconnectivity scores as a function of the number of clusters (Supplementary Fig. 7). To determine the optimal number of clusters, we used NbClust^97^ and searched up to 10 cluster solutions. The 16 indices calculated by the R function NbClust revealed that *k* = 2 (7/16) was the optimal solution. In Supplementary Fig. 7, we also report the number of mouse models grouped in the smallest cluster for increasing *k* (that is, beyond the optimal *k* = 2 partition). This analysis revealed that clusters at *k* = 2 comprised a substantial number of models (*n* = 9 and *n* = 11), ensuring stability and interpretability. Higher partition numbers (that is, *k* = 3 and higher) instead were associated with at least one cluster containing only two models. Considering that (1) NbClust indicated *k* = 2 as optimal partition, (2) our goal was to identify dominant brain topographies that could be robustly translated to human brain scans and (3) partitions with *k* > 3 would be composed of only two mouse models, we retained *k* = 2 for all subsequent analyses.

To rule out that MRI site or anesthesia protocol influenced fMRI connectivity mapping, we assessed the site independence of our results by performing hierarchical clustering on connectivity matrices obtained from wild-type control littermates only (Supplementary Fig. 8). We then quantified the proportion of animals acquired at each site (IIT versus ETH) within the resulting clusters. We found that control littermates were evenly represented across sites in both clusters (IIT: 60%; ETH: 40%), consistent with the composition of the original database (IIT: 12 models; ETH: eight models). Moreover, clustering of wild-type controls did not recapitulate the distribution of etiological hypoconnectivity and hyperconnectivity subtypes, as each wild-type cluster included a balanced proportion of controls from both groups (Supplementary Fig. 8). These analyses suggest that the identified subtypes are not driven by site-dependent factors.

To identify brain regions showing prominent cross-etiological fMRI dysconnectivity in each cluster (that is, subtype), we first thresholded and binarized their global connectivity maps at Cohen’s *d* > 0.8, and then we calculated conjunction maps across autism-relevant mouse models for each subtype. The use of conjunction maps allowed us to identify brain regions that were consistently hypoconnected or hyperconnected in at least two mouse models belonging to the same subtype. These analyses allowed us to generate two distinct conjunction maps, one showing brain regions predominantly hypoconnected (across mouse lines belonging to the hypoconnectivity subtype), the other one showing brain regions predominantly hyperconnected (across mouse lines belonging to the hyperconnectivity subtype).

### Functional network mapping

To investigate the fMRI network atypicalities associated with each subtype, we calculated fMRI connectivity within and between networks by using network-based statistics (NBS)^88^. To this aim, we first extracted fMRI timeseries from 64 cortical and subcortical parcels. Cortical regions were grouped in the nine resting-state functional connectivity networks. We then calculated the ROI-to-ROI functional connectivity matrix for each brain scan by using temporal Pearson’s correlation. We next regressed out the whole-brain mean connectivity signal (that is, mean correlation of each mouse 64 × 64 parcel-to-parcel connectivity matrix) across mouse models. This step allowed us to assess the network organization of each subtype map beyond the general hyperconnectivity and hypoconnectivity characterizing the two autism subtypes. We finally used unpaired *t*-tests as implemented by NBS to identify intergroup functional connectivity differences between autism mouse models and littermates, within each subtype. Statistical results were corrected for multiple comparisons using familywise error rate (FWER). We set the univariate threshold to *t* > 3.1 and the network significance at *P* = 0.05 (two-tailed), and we carried out 5,000 permutations. This analysis yielded a group-level functional connectivity matrix for each subtype showing the absolute number of ROI-to-ROI fMRI connectivity surviving NBS statistical thresholding within and between functional networks. To keep into account that functional networks have a different size (and then are formed by a different number of ROIs), we normalized the functional connectivity matrix by the total number of ROI-to-ROI fMRI connectivity, for within and between functional networks. Networkwise fMRI connectivity differences for each subtype are displayed using connectograms as implemented in the R package ‘circlize’.

### Gene enrichment analysis

To investigate whether different patterns of autism-relevant dysconnectivity reflect dissociable biological pathways, we conducted a gene enrichment analysis. This analysis compares two lists of genes, statistically testing whether a gene set in one list is represented in the other list above chance using a hypergeometric test. Gene enrichment analysis was carried out between a list of autism-relevant genes grouped in the two subtypes of the mouse models (plus their interacting genes) and a list of genes indexing the molecular pathways known to be associated with autism. To this purpose, we created two in silico protein−protein mega-interactomes, one for the hypoconnected subtype and another for the hyperconnected subtype. Each mega-interactome includes all the genes (inferred by the corresponding mouse model) that clustered into either the hypoconnectivity or hyperconnectivity subtype, along with their interacting genes. To search for those interacting genes, we carried out a protein–protein interaction analysis using STRING-DB^98^. For monogenic mouse models (for example, *Shank3* and *Cntnap2*), we used the single mutated or knockout gene as a seed in protein−protein interaction analysis. For 16p11.2 (ref. ^99^) and 22q11.2 (ref. ^100^), we carried out simultaneous seed-based analysis for all the genes included in these two copy number variants (*n* = 27 for 16p11.2 and *n* = 24 for 22q11.2, as modeled in our mice). As an inbred model not relatable to specific genetic alterations, BTBR mice were excluded from this analysis. Overall, these analyses produced a protein−protein interactome of up to 100 genes for each autism etiology. The interactomes of the mouse models within each dysconnectivity subtype were then aggregated to create two mega-interactomes. This analysis produced one mega-interactome of the genes associated with fMRI hypoconnectivity (*n* = 527) and another for those linked to fMRI hyperconnectivity (*n* = 585). We next filtered out genes present in both mega-interactomes (*n* = 70) to retain those uniquely represented in either of the mega-interactomes, resulting in *n* = 457 and *n* = 515 for the hypoconnectivity and hyperconnectivity subtypes, respectively. This step allowed us to focus our subsequent investigations on pathways specifically linked to the two dysconnectivity subtypes. For each interactome, we next applied a gene enrichment analysis to identify the prevalence of molecular pathways known to be dysregulated in autism, as described in ref. ^36^. Molecular pathways included adaptive immune system, chromatin organization, cytokine signaling in immune system, gene expression transcription, innate immune system, MAPK family signaling cascade, membrane trafficking, mTOR signaling, nervous system development, protein−protein interaction at synapses, signaling by GPCR, signaling by WNT, translation and transmission across chemical synapses. The ontologies of the molecular pathways were downloaded from https://reactome.org/. Together with molecular ontologies, we also carried out gene enrichment analysis for a list of manually curated synaptic genes (‘SynGO’)^101^.

For completeness, we report enrichment of the shared gene list in Supplementary Fig. 9. All major pathways (except chromatin organization) were significantly enriched within this pool of shared genes. This is an expected result, as this gene pool largely reflects the etiologies (and autism models) based on which the interactomes were built in the first place. To identify molecular pathways that bias fMRI network changes toward hypoconnectivity or hyperconnectivity, in this study we thus analyzed only the pools of genes specific to each subtype—that is, removing the shared genes represented in our database. This approach is conceptually related to genome-wide association studies: once shared mechanisms are accounted for, distinct biological processes can be identified that differentiate network alterations toward hypoconnectivity or hyperconnectivity.

To probe the robustness of our gene enrichment analysis against interactome size (that is, the maximum number of interacting genes), we repeated the enrichment using interactomes composed of up to 500 genes. This analysis resulted in *n* = 1,793 genes of the interactomes of the hyperconnectivity subtype and *n* = 1,526 genes of the hyperconnectivity subtype. After filtering out the *n* = 377 genes shared by the hypoconnectivity and hyperconnectivity mega-interactomes, we obtained *n* = 1,416 genes uniquely part of the hypoconnectivity subgroup and *n* = 1,149 genes of the hyperconnectivity subgroup. Enrichment analysis conducted with these larger mega-interactomes resulted in ORs highly similar to those obtained with the larger mega-interactomes, thus ruling out that the size of the interactome could drive results (Extended Data Fig. 2). We obtained analogous enrichments when we reduced the network of interacting genes to 50, 25 or 10 (Extended Data Fig. 2). Finally, a complementary enrichment analysis was carried out between the meta-interactomes and the genes of the co-expression modules reported to be differentially expressed in postmortem brain tissues of individuals with autism^16^. Only co-expression modules of known biological function (*n* = 10^16^) were considered for this analysis. Enrichments were measured by using hypergeometric statistical testing and quantified with ORs and *P* values (FDR corrected at *q* < 0.05). The gene lists and the interactomes are listed in Supplementary Table 2.

### Human studies

#### Sample

To identify autism subtypes informed by the biologically relevant rodent dysconnectivity subtypes, we analyzed resting-state fMRI timeseries from *n* = 940 individuals with idiopathic autism and *n* = 1,036 neurotypical controls comprising 38 data collections, 37 selected from the two ABIDE data repositories^39,40^ and an additional one more recently aggregated at the CMI^41,42^. Ethical approval was obtained at each contributing site, and informed consent and/or assent were obtained for participants in accordance with local institutional review board/ethics committee requirements. We included data from individuals aged 5−30 years. As illustrated in the data selection flow in Supplementary Fig. 10, we retained only brain scans of participants with median framewise displacement ≤ 0.2 mm (ref. ^102^) that successfully underwent co-registration to MNI standard space. Details on each collection, including scan parameters, are at http://fcon_1000.projects.nitrc.org/indi/abide/abide_I.html for ABIDE I and at http://fcon_1000.projects.nitrc.org/indi/abide/abide_II.html for ABIDE II. Details on the CMI data collection are reported in refs. ^41,42^. Demographics and clinical information of the aggregate sample included in our analyses are summarized in Table 2.

### Resting-state fMRI preprocessing

Before mapping fMRI connectivity, we preprocessed fMRI timeseries with C-PAC version 1.6.2 (ref. ^103^). In brief, we resampled the data to right−posterior−inferior (RPI) orientation (AFNI 3drefit and AFNI 3dresample) and conducted slice timing correction (AFNI 3dTshift). Next, motion correction (AFNI 3dvolreg) was performed using a two-stage approach in which the timeseries were first co-registered to the temporal mean fMRI image, and then a new temporal mean was calculated and used as the target for a second co-registration^104^. At this second stage, motion parameters based on the Friston 24-parameter model (six motion parameters, their first-order derivatives and 12 squared values of these items) were calculated along with framewise displacement. Motion-corrected timeseries were then skull stripped (AFNI 3dAutomask) and mean-based intensity normalized to a factor of 10,000. Then, nuisance variable regression was performed with a 24-regressor model of motion and five nuisance signals, identified via principal component analysis of signals obtained from white matter and mean cerebrospinal fluid (CSF) signal (CompCor, ref. ^105^). Brain mask (probability *P* > 0.95) of white matter and CSF were calculated by applying FSL’s FAST tool to co-registered structural MRI images^106^. Functional-to-anatomical co-registration was achieved by boundary-based registration using FSL FLIRT. The residuals of the nuisance variable regression procedure were then processed with bandpass filtering (0.01 Hz < *f* < 0.1 Hz) and subsequently smoothed using a 6-mm FWHM kernel. Finally, spatial normalization of preprocessed fMRI timeseries to MNI152 space was applied with linear and nonlinear registration using ANTs^107^.

### Resting-state fMRI connectivity mapping

For consistency with mouse data, fMRI connectivity was quantified by applying the same global connectivity mapping described above^95^. Global connectivity maps were then harmonized across all data collections (*n* = 38) using ComBat (ref. ^108^). Before carrying out subtyping, the fMRI global connectivity map of participants with autism underwent voxelwise *z*-scoring normalization against the mean and standard deviation of the fMRI connectivity maps of the neurotypical participants to interpret connectivity patterns of the participants with a diagnosis of autism in terms of connectivity increases or decreases relative to neurotypical participants.

### Autism subtyping using fMRI connectivity

We used normalized global connectivity maps to discover subtypes of autism based on the cross-species regional approach summarized in Fig. 3. Specifically, we searched for normalized global connectivity maps of individuals with autism showing patterns of fMRI hypoconnectivity or hyperconnectivity corresponding to those we observed in the mouse subtypes. To establish regional correspondence across species, we selected *n* = 13 cortical and subcortical regions for which plausible anatomical and functional rodent precursors were previously described^23,109–111^. These include associative and limbic cortices, such as the medial prefrontal, anterior and mid-cingulate and retrosplenial cortices (that is, cytoarchitecturally conserved components of the mouse default mode network^91,112–114^, the insular cortex^91,115^ and primary somatosensory areas (somatosensory, auditory and visual cortices^110^)). Subcortical structures included the striatum, thalamus, amygdala and hypothalamus^110^ and hippocampus^116,117^. Boundaries of anatomical regions for the mouse brain were defined by using the Allen Mouse Brain Reference Atlas, and those of the human brain were defined by the Harvard Oxford Atlas (Supplementary Fig. 2a). A more detailed account of the pipeline that we used for our cross-species subtyping with fMRI is depicted in Supplementary Fig. 1. We first used the conjunction maps of each of the two mouse subtypes to generate ‘dysconnectivity priors’ for hypoconnectivity and hyperconnectivity. For each conjunction map, we measured the percentage of voxels exhibiting hypoconnectivity (or hyperconnectivity) in each of the *n* = 13 regions (Cohen’s *d* > 0.8). By applying *k*-means clustering to those percentages among the 13 regions, we then separated brain regions with more prominent dysconnectivity from those with less prominent dysconnectivity (hypo or hyper). This strategy led us to the identification of *n* = 5 brain regions with prominent hypoconnectivity—namely, anterior and middle cingulate, insula, motor cortex and striatum (Supplementary Fig. 2b). These regions were aggregated into an aggregated mouse hypoconnectivity mask. For the rodent hyperconnectivity subtype, we identified *n* = 3 brain regions with prominent hyperconnectivity, namely the amygdala, hippocampus and striatum (Supplementary Fig. 2b), which we aggregated into a mouse hyperconnectivity mask as above. We next used these two masks to guide the identification of hypoconnectivity and hyperconnectivity subtypes in the human dataset. Participants on the autism spectrum exhibiting fMRI connectivity lower than 1 s.d. in the regions belonging to the hypoconnectivity mask were grouped into the hypoconnectivity subtype (Supplementary Fig. 1). Similarly, participants with a diagnosis of autism exhibiting fMRI connectivity higher than 1 s.d. in the set of regions homologous to the rodent hyperconnectivity mask were grouped into the hyperconnectivity subtype (Supplementary Fig. 1). To rule out that subtypes could be differentiated by in-scanner head motion, we measured median framewise displacement for each individual across the two subtypes. Group mean comparisons confirmed similarly low levels of head motion across the subtypes (Supplementary Table 1).

### Replicability of subtyping

To test the replicability of our fMRI subtypes, we a priori split the cohort of individuals with autism into a discovery dataset and a replication dataset and searched for subtypes in both datasets independently using the same method. To create the discovery and replication datasets, we used the R function ‘group_by’, by following the conditions that (1) the two datasets must be carefully matched by diagnosis, sex, age and in-scanner head motion and (2) one dataset comprises at least 70% of the sample. The resulting matched datasets comprised 78.5% of the brain scans in the discovery dataset (*n* = 744 participants with autism, *n* = 807 neurotypical participants, *n* = 38 data collections) and 21.5% in the replication dataset (*n* = 196 participants with autism, *n* = 229 neurotypical participants, *n* = 38 data collections). Matching between discovery and replication datasets was confirmed by unpaired *t*-tests for the continuous variables age (*t*_1,974_ = 1.09, *P* = 0.28) and in-scanner head motion (*t*_1,974_ = 0.10, *P* = 0.92) and by *χ*^2^ for the categorical variables, such as diagnosis (*χ*_1_^2^ = 0.45, *P* = 0.49) and sex (*χ*_1_^2^ = 1.82, *P* = 0.18). Global connectivity scores of the individuals with autism grouped in the hypoconnected and hyperconnected subtypes were then *z*-scored relative to the neurotypical group distribution for the discovery and replication datasets together. *z*-scored global connectivity values were then quantified in the 13 evolutionarily conserved regions (Supplementary Fig. 2c,d).

Replicability was measured with spatial similarity metrics between the fMRI brain maps of the two subtypes identified in the discovery and replication datasets. Specifically, we quantified Dice coefficients between conjunction maps, obtained after thresholding and binarizing the fMRI connectivity maps of each subtype at *t* = 3.1 (FWER cluster corrected at *P* < 0.05) in the discovery and replication datasets (hypoconnectivity subtype: Supplementary Fig. 3a; hyperconnectivity subtype: Supplementary Fig. 3c). Replicability was also assessed by computing spatial correlation (Pearson’s *r*) of unthresholded maps (hypoconnectivity subtype: Supplementary Fig. 3b; hyperconnectivity subtype: Supplementary Fig. 3d). Finally, to rule out that the subtyping could be biased by brain scans acquired in a small fraction of laboratories, we repeated the subtyping described above in the aggregated sample after removing the data collections containing the largest number of participants with autism.

### Functional network mapping

To investigate the fMRI network atypicalities associated with each subtype, we calculated fMRI connectivity within and between networks by using NBS (ref. ^88^). To this aim, we first extracted fMRI timeseries from 400 cortical parcels of the Schaefer atlas^44^ and the 14 subcortical parcels of the Harvard Oxford Atlas^45^, distributed with FSL. Schaefer cortical regions were grouped in the seven canonical resting-state functional connectivity networks described in ref. ^87^. We then calculated the ROI-to-ROI functional connectivity matrix for each brain scan by using temporal Pearson’s correlation. We next regressed out the whole-brain mean connectivity signal (that is, mean correlation of each participant’s 414 × 414 parcel-to-parcel connectivity matrix) across participants. This step allowed us to assess the network organization of each subtype map beyond the general hyperconnectivity and hypoconnectivity characterizing the two autism subtypes. We finally used unpaired *t*-tests as implemented in NBS to identify intergroup functional connectivity differences between individuals with autism and neurotypical individuals, within each subtype. Statistical results were corrected for multiple comparisons using FWER. We set the univariate threshold to *t* ≥ 3.1 and the network significance at *P* = 0.05 (two-tailed), and we carried out 5,000 permutations. This analysis yielded a group-level functional connectivity matrix for each subtype showing the absolute number of ROI-to-ROI fMRI connectivity surviving NBS statistical thresholding within and between functional networks. To keep into account that functional networks have a different size (and then are formed by a different number of ROIs), we normalized the functional connectivity matrix by the total number of ROI-to-ROI fMRI connectivity, for within and between functional networks. Networkwise fMRI connectivity differences for each subtype are displayed using connectograms as implemented in the R package ‘circlize’.

### Behavioral analyses

To investigate whether the autism connectivity subtypes could be differentiated by autism behavioral phenotypes, we used ratings consistent with ADOS-2. We used ADOS as primary measures because of its clinical validity, specificity and wide use^118,119^. We focused on total CSSs^120,121^. Furthermore, to assess potential variations in the symptoms contributing to total CSS, we also examined the CSSs of the subdomain scales ‘social affect’ and RRB^122^. CSSs range from 1 to 10, with higher scores indicating greater severity; they allow comparability across ADOS modules, which vary by age and language abilities^120–122^. We used these scores to describe the aggregate sample and for group mean comparisons of the identified autism-related brain dysconnectivity subtypes.

For ABIDE data collected and aggregated prior to the publication of ADOS-2 (ref. ^119^), total CSSs were computed within each data collection’s site based on corresponding algorithms on Autism Diagnostic Observation Schedule-Generic (ADOS-G) relevant items per Gotham et al.^121^ (for modules 2 and 3) and Hus and Lord^120^ (for module 4). As a result, in the present study, total CSSs were available for *n* = 549 (58.4%) of the 940 data with an autism diagnostic label included in the aggregated sample. These included all *n* = 63 data from CMI, a subset of the ABIDE I (*n* = 9 data collections: KKI, NYU, UCLA-1, UCLA-2, UM-1, UM-2, USM, Stanford and Yale) and a subset of the ABIDE II (*n* = 11 data collections: GU-1, IP-1, KKI-1, NYU-1, NYU-2, OHSU-1, SDSU-1, SU-2, U-MIA-1, UCD-1 and UCLA-1). To examine the role of autism phenotypic subdomains, we converted available social affect and RRB subscale scaled scores into corresponding CSSs based on Hus and Lord^53^ guidelines. Social affect scores were available for *n* = 520 (55.3%) individuals in the autism group. RBB scores were available for *n* = 525 (55.8%) individuals in the autism group. Unpaired *t*-tests (FDR corrected for multiple comparisons at *q* ≤ 0.05) were used to assess subtype group mean differences in behavioral scores. Secondary analyses explored subtype comparisons in regard to demographics, IQ scores, rate of psychiatric co-occurrences and psychoactive medication use available (Supplementary Table 1).

To examine whether the fMRI maps of the subtypes exhibit spatial topography similar to those of the brain maps associated with cognitive functions, we used the 12 ontology probability maps identified in ref. ^43^. Consistent with ref. ^123^, spatial correspondence was measured as the proportion (%) of voxels by which each neurocognitive map thresholded at *P* < 1 × 10^−5^ overlaps with the mean regressed fMRI maps of each of the subtypes at Cohens’ *d* ≥ 0.2. The resulting spatial correspondences were visualized with radar plots.

### Brain decoding and gene enrichment analyses

To search for an autism-relevant genetic signature in the fMRI connectivity maps of the two autism subtypes, we carried out brain decoding and gene enrichment analysis. The goal of these analyses is to test whether genes exhibiting expression patterns spatially correlated to the fMRI maps of the two subtypes are differentially expressed in autism^16^. To identify genes that are spatially enriched in the dysconnectivity patterns characteristic of each subtype, we carried out brain decoding with NeuroVault^124,125^. The analysis first uses a mixed linear model to compute the similarity between an unthresholded whole-brain fMRI map and spatial patterns of gene expression for each of the six donor brains of the Allen Institute Human Brain Gene Expression Atlas^125^. The slopes of these donor-specific linear models encode how similar each gene’s spatial expression pattern is to the fMRI map. Donor-specific slopes were then subjected to a one-sample *t*-test to identify genes whose spatial expression patterns show consistently high similarity across the donor brains to the fMRI map. The resulting list of genes is then thresholded for multiple comparisons, and only the genes with *t*-statistic values surviving FDR *q* < 0.05 are retained for the following enrichment analysis. To increase specificity of the spatial similarity between gene expression and fMRI brain maps of the two autism subtypes, we retained only genes highly expressed in the brain (*n* = 16,796 genes). This analysis resulted in a list of *n* = 3,840 genes for the hypoconnectivity subtype and *n* = 4,463 genes for the hyperconnectivity subtype (FDR corrected, *q* < 0.05) (Supplementary Table 3). We next filtered out genes present in both lists (*n* = 2,193) to retain those uniquely represented in either list. This resulted in *n* = 1,647 and *n* = 2,270 for the hypoconnectivity and hyperconnectivity subtypes, respectively. This step allowed us to focus our subsequent investigations on pathways uniquely associated with each dysconnectivity subtype. With the lists of fMRI-relevant genes isolated, we then carried out a set of gene enrichment analysis to examine whether our fMRI transcriptomic signatures (that is, the brain decoded genes) were enriched for specific molecular pathways. The first of these sets comprises genes differentially expressed in autism. This list was obtained by aggregating all the genes belonging to the *n* = 24 co-expression modules reported to be differentially expressed in postmortem brain tissues of individuals with autism^25^. A second gene set encompassed molecular pathways known to be dysregulated in autism as described in ref. ^23^. This was generated by downloading (from https://reactome.org/) the ontologies of autism-relevant human pathways homologous of those we investigated for the mouse brain. Here, we also included a gene enrichment analysis for a list of manually curated synaptic genes (‘SynGO’)^24^. We finally probed enrichment for a third set of genes this time encompassing gene co-expression modules labeled with known biological function (*n* = 10^25^). Enrichment was measured by using hypergeometric statistical testing and quantified with ORs and *P* values. Genes surviving correction for multiple comparisons at FDR *q* < 0.05 were considered statistically significant. A list of brain decoded genes and pathways is reported in Supplementary Table 3.

### Reporting summary

Further information on research design is available in the Nature Portfolio Reporting Summary linked to this article.

## Online content

Any methods, additional references, Nature Portfolio reporting summaries, source data, extended data, supplementary information, acknowledgements, peer review information; details of author contributions and competing interests; and statements of data and code availability are available at 10.1038/s41593-026-02287-z.

## Supplementary information

Supplementary Information Supplementary Figs. 1−10 and Supplementary Table 1.

Reporting Summary

Supplementary Table 2 Protein−protein interactomes and gene lists used in enrichment analyses in the mouse models.

Supplementary Table 3 Brain decoded genes and gene lists used in enrichment analyses in humans.

Supplementary Table 4 Gene lists used in enrichment analysis for other brain conditions.

## Source data

Source Data Source data of all figures.

## Data Availability

Raw mouse fMRI timeseries can be download from https://dataverse.iit.it/ (10.48557/AIO2LN). Human fMRI scans from ABIDE I and ABIDE II scans are available at https://fcon_1000.projects.nitrc.org/indi/abide/abide_I.html and https://fcon_1000.projects.nitrc.org/indi/abide/abide_II.html. Most CMI-based datasets are deposited in the National Database for Autism Research (NDAR) (10.15154/nnfr-4943) upon parent/legal guardian consent and are accessible through the NDAR (https://ndar.nih.gov/) in accordance with its data use policies. Source data are provided with this paper.

## Code availability

The code used for preprocessing mouse fMRI data is available at https://github.com/functional-neuroimaging/rsfMRI-preprocessing. The code for mapping global connectivity in mice and humans is available at https://github.com/functional-neuroimaging/rsfMRI-global-local-connectivity. The code employed for gene enrichment analysis is available at https://github.com/functional-neuroimaging/gene_decoding_and_enrichment. The code for site harmonization and cluster analysis is available at https://github.com/functional-neuroimaging/biological_subtyping.
