# Supplementary material for: Autism subtypes identified using cross-species functional connectivity analyses
Source: Nat Neurosci. 2026 May 15;29(6):1476–87. doi: 10.1038/s41593-026-02287-z (PMC13246445; doi:10.1038/s41593-026-02287-z)
Supplement: Supplementary file 1 — Supplementary Figs. 1−10 and Supplementary Table 1. [file 41593_2026_2287_MOESM1_ESM.pdf]

nature neuroscience

Supplementary information https://doi.org/10.1038/s41593-026-02287-z

Autism subtypes identified using cross-species functional connectivity analyses

In the format provided by the authors and unedited

Supplementary Material

Autism subtypes identified using cross-species functional connectivity analyses

Marco Pagani[1,2,3], Valerio Zerbi[4,5], Silvia Gini[1,6], Filomena Alvino[1], Abhishek Banerjee[7], Andrea Barberis[8], M. Albert Basson[9,10], Yuri Bozzi[6], Alberto Galbusera[1], Jacob Ellegood[11], Michela Fagiolini[12], Jason Lerch[13], Michela Matteoli[14,15], Caterina Montani[1,+], Davide Pozzi[15], Giovanni Provenzano[16], Maria Luisa Scattoni[17], Nicole Wenderoth[18], Ting Xu[19], Michael Lombardo[20], Michael P Milham[21], Adriana Di Martino[2]*, Alessandro Gozzi[1]*

[1]Functional Neuroimaging Laboratory, Istituto Italiano di Tecnologia, Center for Neuroscience and Cognitive Systems, CNCS@UNITN, Rovereto, Italy
[2]Autism Center, Child Mind Institute, New York, NY, USA
[3]IMT School for Advanced Studies, Lucca, Italy
[4]Department of Psychiatry, University of Geneva, Switzerland
[5]Department of Basic Neurosciences, University of Geneva, Switzerland
[6]Center for Mind and Brain Sciences (CIMeC), University of Trento, Rovereto, Italy
[7]Brain Research Institute, University of Zurich, Zurich, Switzerland
[8]Synaptic Plasticity of Inhibitory Networks, Istituto Italiano di Tecnologia, Genova, Italy
[9]Centre for Craniofacial and Regenerative Biology, King's College London, London, UK
[10]Department of Clinical and Biomedical Sciences, University of Exeter, Exeter, UK
[11]Bloorview Research Institute, Holland Bloorview Kids Rehabilitation Hospital, Toronto, ON, Canada
[12]Boston Children's Hospital, Harvard Medical School, Boston, MA, USA
[13]Wellcome Centre for Integrative Neuroimaging, FMRIB, Nuffield Department of Clinical Neurosciences, University of Oxford, Oxford, UK
[14]Humanitas University, Milan, Italy
[15]CNR Institute of Neuroscience c/o Humanitas Clinical and Research Center - IRCCS, Rozzano, Milan, Italy
[16]Department of Cellular, Computational and Integrative Biology. University of Trento, Trento, Italy
[17]Research Coordination and Support Service, Istituto Superiore di Sanità, Rome, Italy
[18]Neural Control of Movement Lab, ETH Zürich, Switzerland
[19]Center for Integrative Developing Brain, Child Mind Institute, New York, NY, USA
[20]Laboratory for Autism and Neurodevelopmental Disorders, Istituto Italiano di Tecnologia, Center for Neuroscience and Cognitive Systems, Rovereto, Italy
[21]Center for the Integrative Developmental Neuroscience, Child Mind Institute, New York, NY, USA

+ Current address: IRCCS Ospedale Policlinico San Martino, Genova, Italy
* These authors contributed equally
Correspondence: Alessandro Gozzi PhD, alessandro.gozzi@iit.it

Supplementary Methods

Supplementary Figures

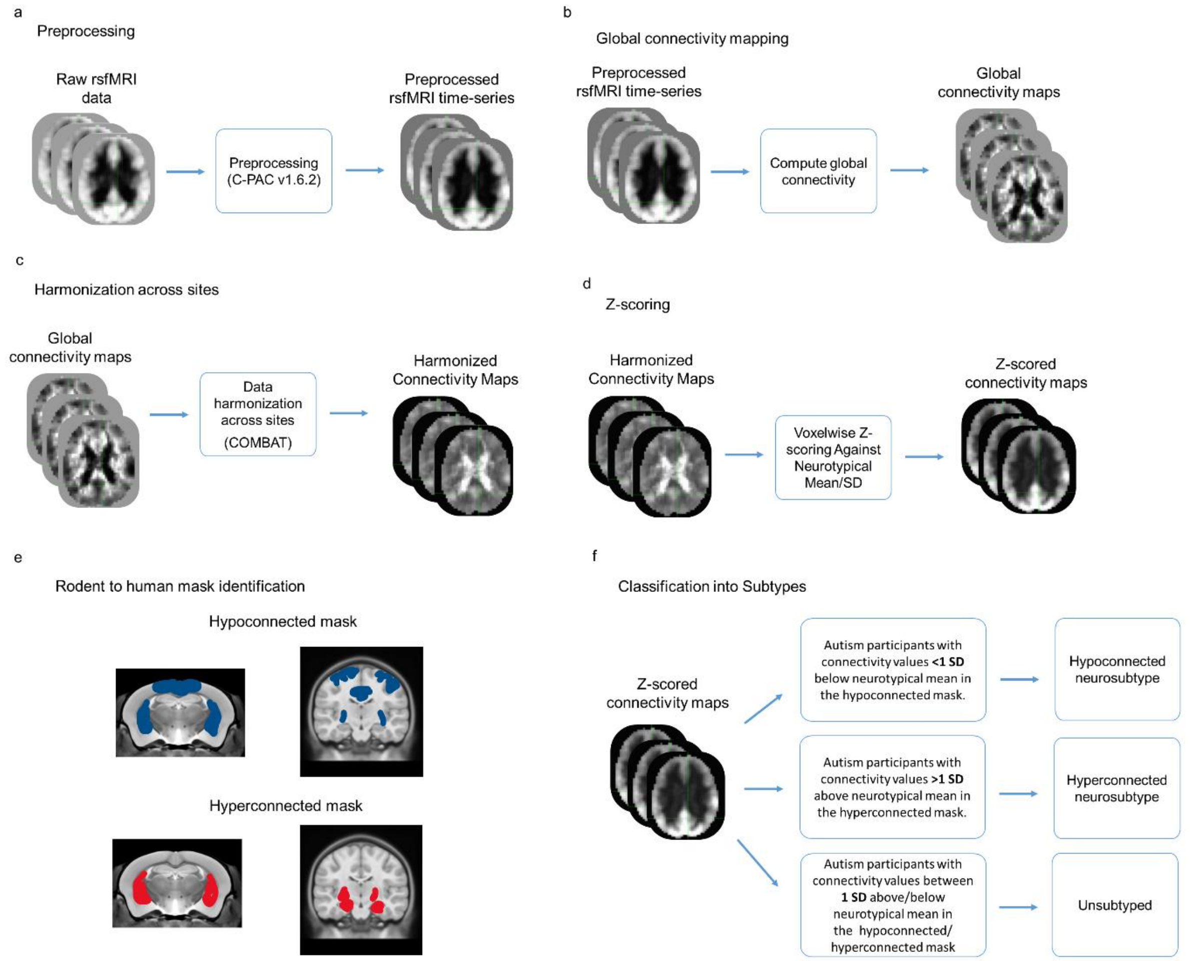

Supplementary Figure 1. Schematic of fMRI autism subtyping guided by rodent findings. a) Raw fMRI data from autistic and neurotypical individuals (NTs) were preprocessed using C-PAC v1.6.2. b) Preprocessed fMRI timeseries were used to compute global connectivity maps for autism and NTs data. c) Global connectivity maps were harmonized across data n=38 data collections with ComBat [6]. d) Harmonized global connectivity maps of autism were normalized with Z-scoring against NTs mean and standard deviation. e) For each mouse model subtype, we identified a subset of 13 evolutionarily conserved regions (Supplementary Figure 3) that best represent predominant dysconnectivity patterns observed across autism-related models. Those regions were combined to generate mouse hypoconnectivity and hyperconnectivity masks. Corresponding hypoconnectivity and hyperconnectivity masks encompassing the same set of brain regions were then created in human data. f) Brain scans of autistic individuals were next grouped into subtypes based on the dysconnectivity patterns computed within the masks described above. Autistic individuals with mean global connectivity values in the hypoconnectivity mask more than 1 SD below those of the NTs were assigned to the hypoconnectivity subtype. Those with values more than 1 SD above in the hyperconnectivity mask were assigned to the hyperconnectivity subtype. All the remaining data were considered “unsubtyped”.

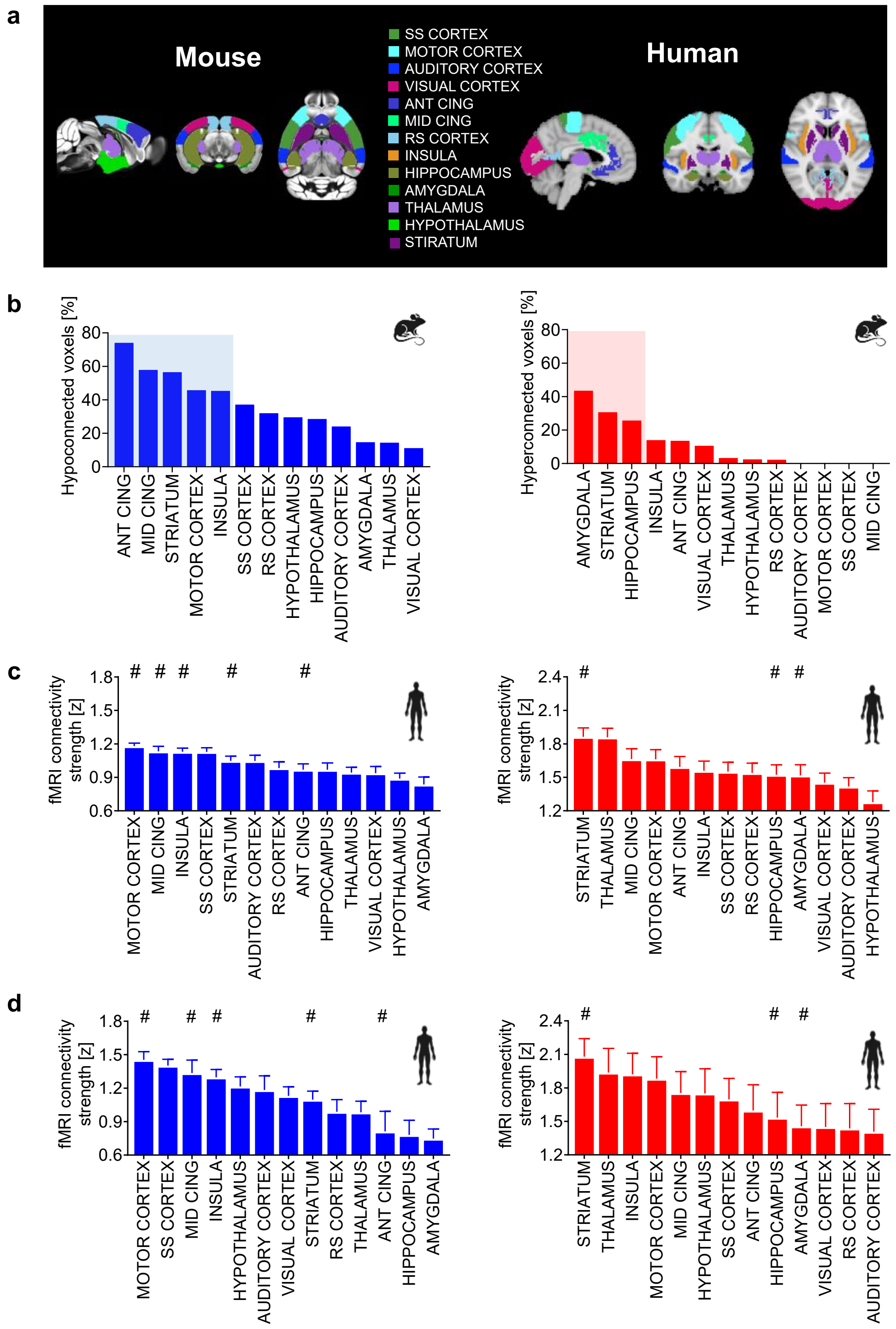

Supplementary Figure 2. Regional quantification of fMRI connectivity in evolutionarily conserved brain regions in the mouse and human brain. a) Illustration of the 13 evolutionarily conserved anatomical regions we selected for cross-species extrapolation of fMRI findings. Homologous regions are depicted with the same color. **b)** Regional quantification of the percentage of voxels exhibiting altered connectivity in the mask of the hypo- (left panel) and hyperconnectivity (right panel) rodent subtypes. Bars represent the proportion

of voxels exhibiting hypo- (left) or hyperconnectivity (right). Mouse brain regions exhibiting the highest dysconnectivity (shaded in blue or red) were merged together to produce a mask that we later used as a “dysconnectivity prior” for cross-species subtyping. **c)** Regional quantification of atypical connectivity in autism hypo- (left) and hyperconnectivity (right) subtypes in the discovery dataset. **d)** Regional quantification of atypical connectivity of the autism hypo- (left) and hyperconnectivity (right) subtypes in the replication dataset. Error bars indicate mean and SEM. # indicates brain regions homologous to those belonging to the corresponding rodent dysconnectivity prior (i.e. those shaded in b). ANT CINGULATE, anterior cingulate; MID CING, middle cingulate; RS CORTEX, retrosplenial cortex; SS CORTEX, somatosensory cortex.

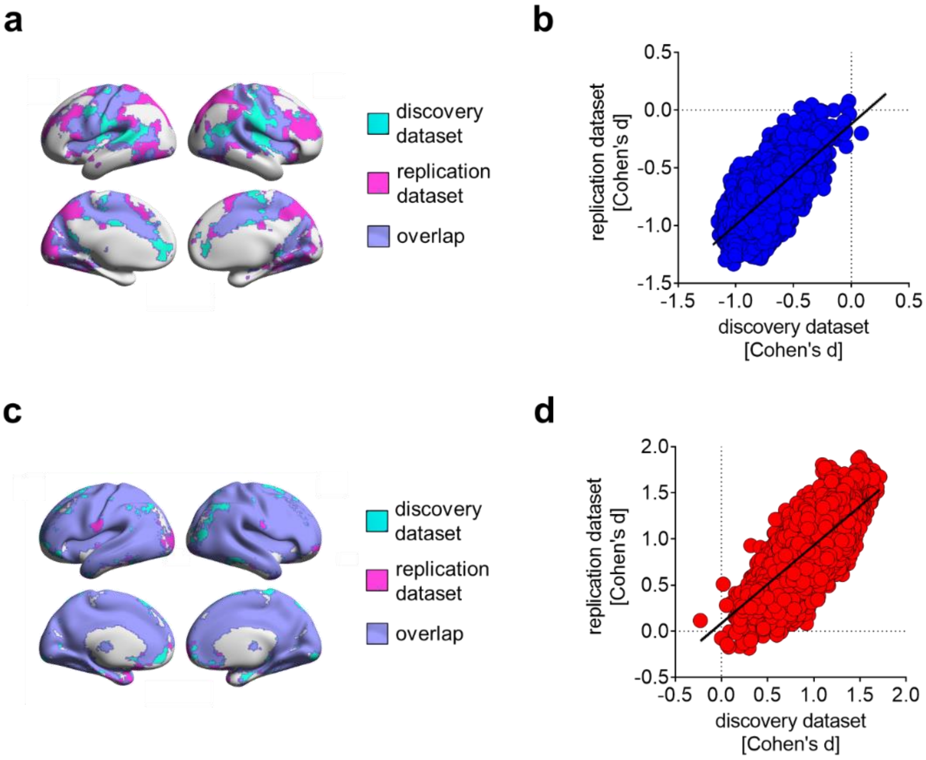

Supplementary Figure 3. Autism subtypes are replicable. a) Spatial overlap of hypoconnectivity subtype maps obtained independently in the discovery and replication autism datasets selected *a priori* from the aggregated sample (Dice coefficient=0.74). Light blue indicates regions exhibiting hypoconnectivity uniquely in the discovery dataset. Purple indicates regions exhibiting hypoconnectivity uniquely in the replication dataset. Violet indicates regions exhibiting hypoconnectivity in both datasets (labelled as “overlap”). **b)** Voxelwise spatial correlation between fMRI connectivity maps of discovery and replication datasets in the hypoconnectivity subtype (Pearson’s r=0.67). Each circle represents a voxel. **c)** Spatial overlap of hyperconnectivity subtype maps obtained, independently, in the discovery and replication datasets (Dice coefficient=0.96). **d)** Voxelwise spatial correlation between fMRI connectivity maps of discovery and replication datasets in the hyperconnectivity subtype (Pearson’s r=0.73). Light blue indicates regions exhibiting hyperconnectivity uniquely in the discovery dataset. Purple indicates regions exhibiting hyperconnectivity uniquely in the replication dataset. Violet indicates regions exhibiting hyperconnectivity in both datasets (labelled as “overlap”).

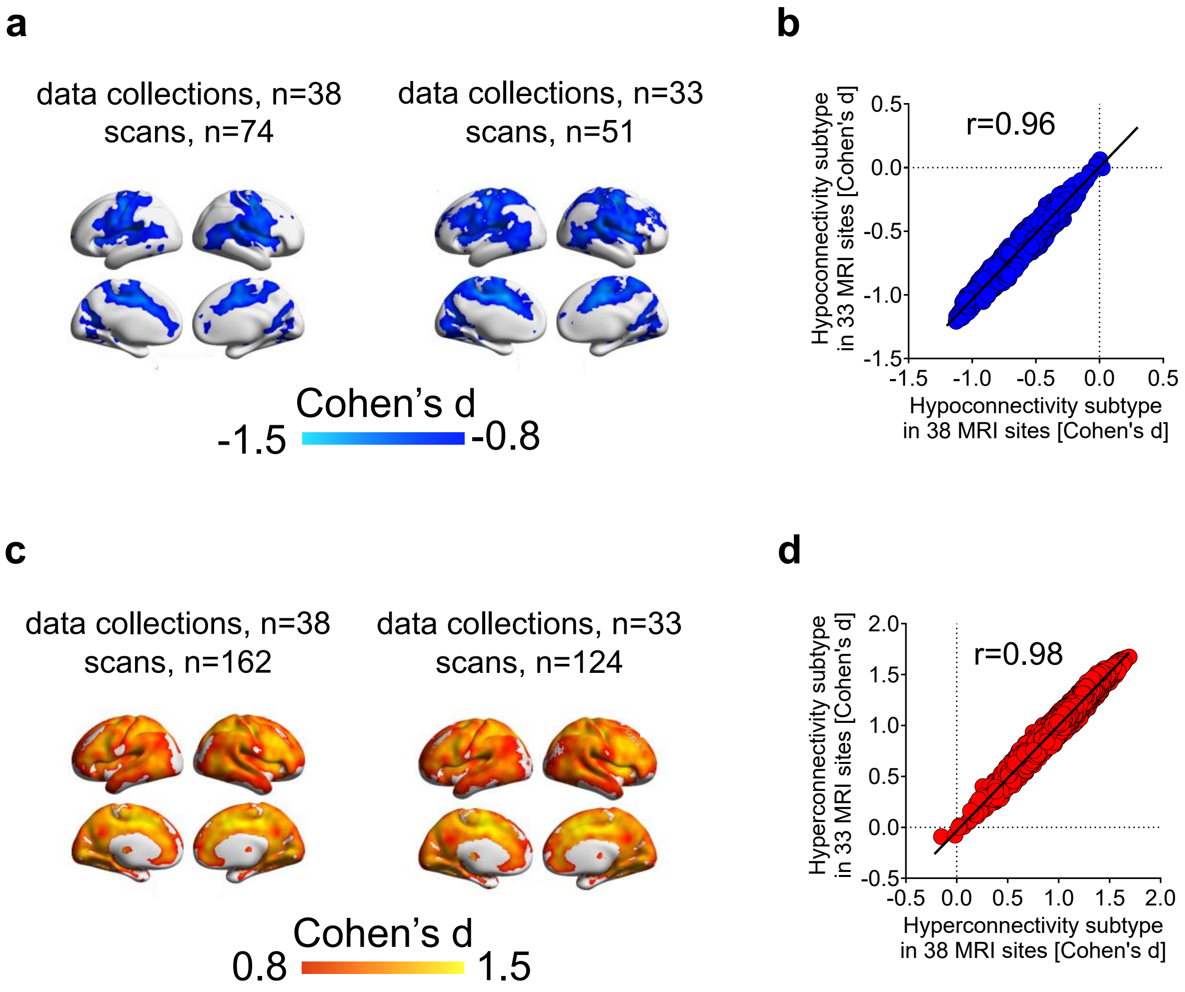

Supplementary Figure 4. Atypical connectivity in the hypo- and hyperconnectivity subtypes is not driven by the largest autism data collections. a) Hypoconnectivity subtype maps obtained using all available n=38 data collections (n=74 hypoconnectivity scans, left panel), or upon exclusion of the scans included in the n=5 data collections with the largest autism datasets (n=51 hypoconnectivity scans, right panel). The n=5 data collections are ABIDEI-NYU (n=76 ASD), CMI (n=63 ASD), ABIDEII-NYU-1 (n=47 ASD), ABIDEII-KKI-1 (n=45 ASD) and ABIDEI-USM (n=40 ASD). **b)** Voxelwise spatial correlation between the two hypoconnectivity maps (Pearson's r=0.96). **c)** Hyperconnectivity subtype maps obtained using all available n=38 data collections (n=162 hyperconnectivity scans, left panel), or upon exclusion of the scans included in the n=5 data collections with the largest sample size (n=124 hyperconnectivity scans, right panel). **d)** Voxelwise spatial correlation between the two hyperconnectivity maps (Pearson's r = 0.98).

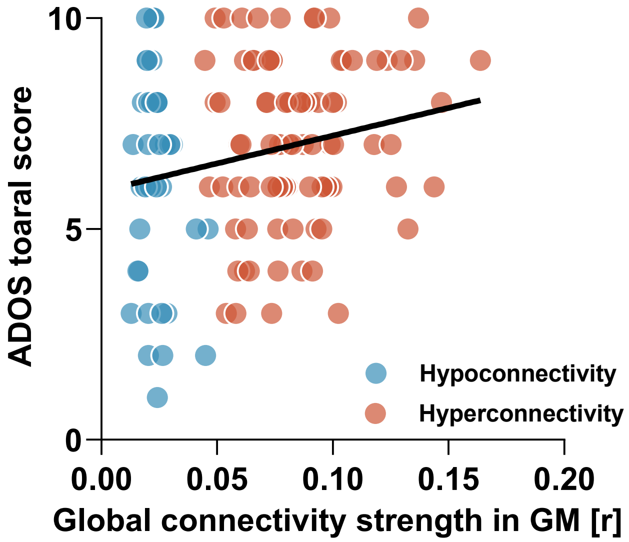

Supplementary Figure 5. Functional connectivity strength in grey matter (GM) scales with ADOS scores across the two subtypes. Mean fMRI connectivity in grey matter areas is significantly correlated with ADOS scores of the autistic individuals grouped in the hypo and hyperconnected subtype (Spearman's rho = 0.18, p = 0.043).

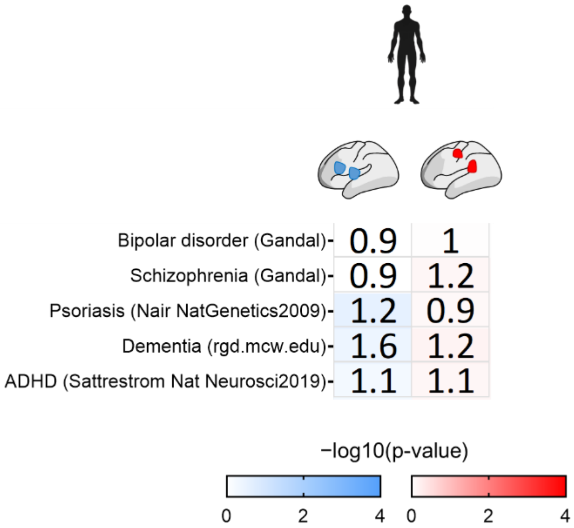

Supplementary Figure 6. Additional gene enrichment analyses. The two subtypes did not show significant enrichment for genes associated with bipolar disorders (hypoconnectivity, OR=0.9, $p_{(FDR)}$ = 0.96; hyperconnectivity, OR=1, $p_{(FDR)}$ = 0.93), schizophrenia (hypoconnectivity, OR=0.9, $p_{(FDR)}$ = 0.99; hyperconnectivity, OR=1.2, $p_{(FDR)}$ = 0.67), psoriasis (hypoconnectivity, OR=1.2, $p_{(FDR)}$ = 0.25; hyperconnectivity, OR=0.9, $p_{(FDR)}$ = 0.82), dementia (hypoconnectivity, OR=1.6, $p_{(FDR)}$ = 0.36; hyperconnectivity, OR=1.2, $p_{(FDR)}$ = 0.58), or ADHD (hypoconnectivity, OR=1.1, $p_{(FDR)}$ = 0.51; hyperconnectivity, OR=1.1, $p_{(FDR)}$ = 0.66). ADHD: attention deficit and hyperactivity disorder. These gene lists are reported in **Supplementary Table 4.**

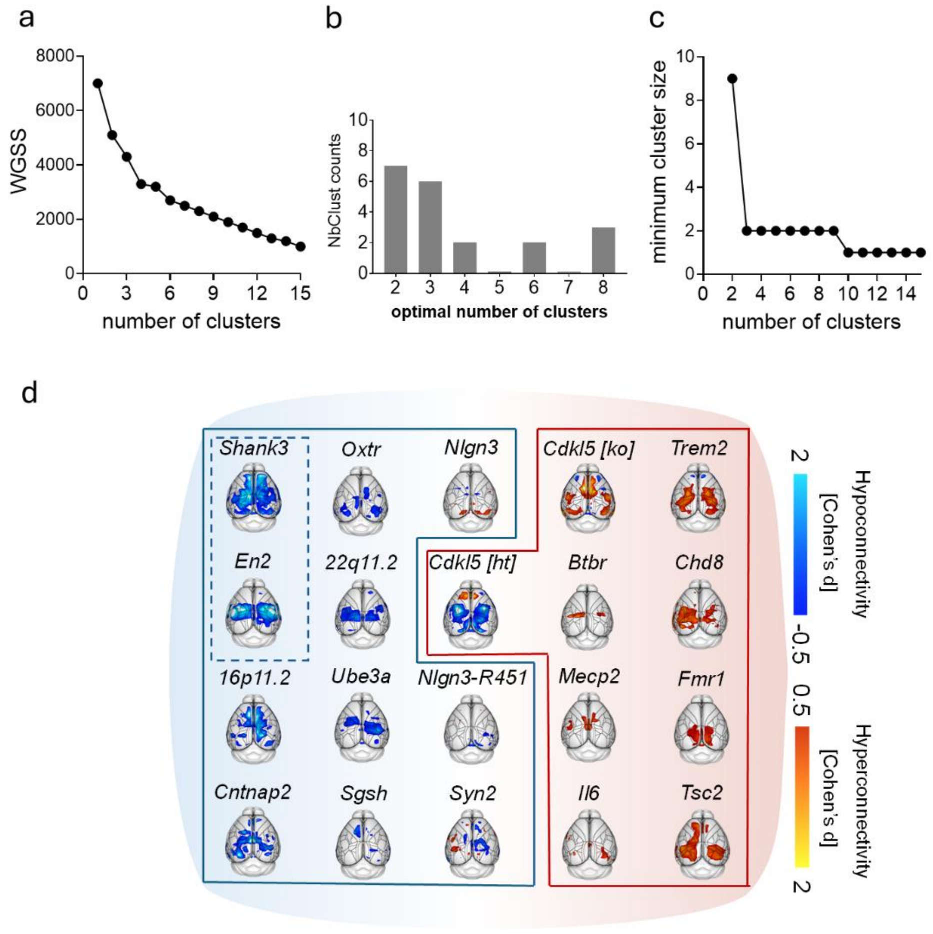

Supplementary Figure 7. Autism mouse models cluster into two dominant subtypes. a) Scree plots indicating within group sum of square (WGSS), as a function of number of clusters. b) NbClust indicates that the optimal clustering solution is k=2. c) Minimum cluster size in terms of number of mouse models for 2<k<9. This value drops to n=2 models for k>3. d) Continuous blue and red lines mark the genetic models grouped in the hypo-connected and hyper-connected subtype. Dashed blue lines indicate the two genetic models clustered with k=3.

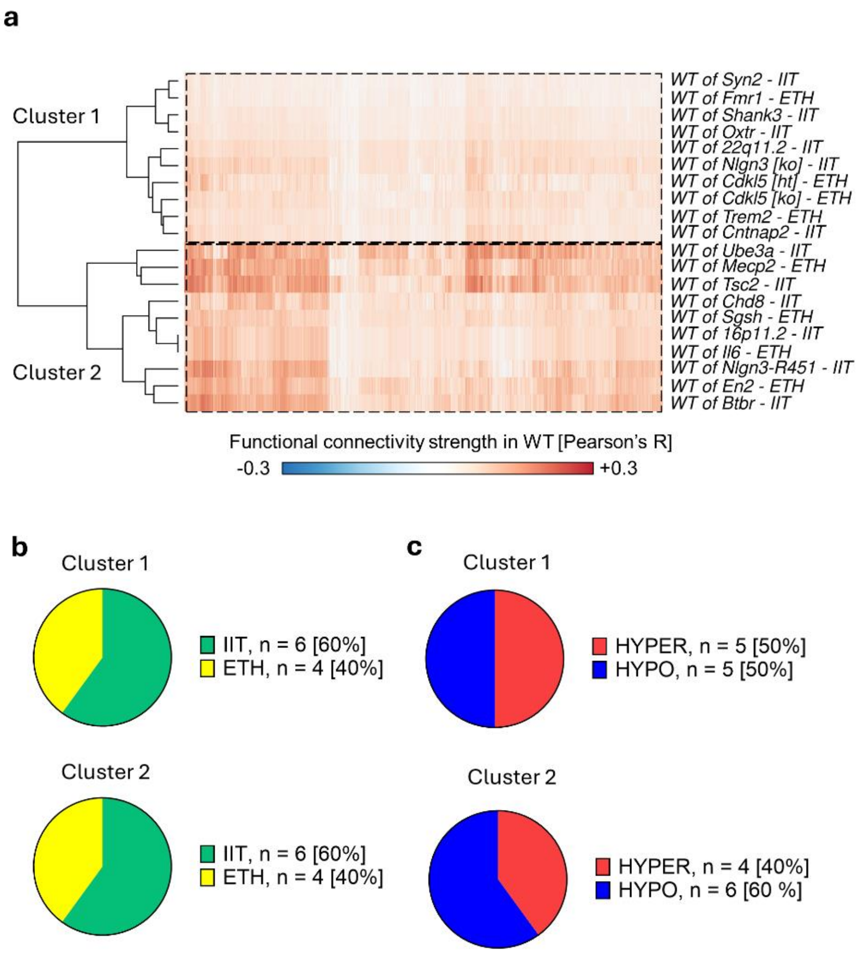

Supplementary Figure 8. Hierarchical clustering of wild type (WT) control mice across IIT and ETH sites. a) Hierarchical cluster analysis of wild type animals based on global fMRI connectivity strength. b) WT mice from the two imaging sites (IIT or ETH) were evenly distributed across the two WT clusters, indicating no site-related grouping. c) WT mice of hypo- and hyperconnectivity associated models were also similarly distributed across the two clusters, showing no systematic association between wild type grouping and the connectivity subtype of the corresponding mutants

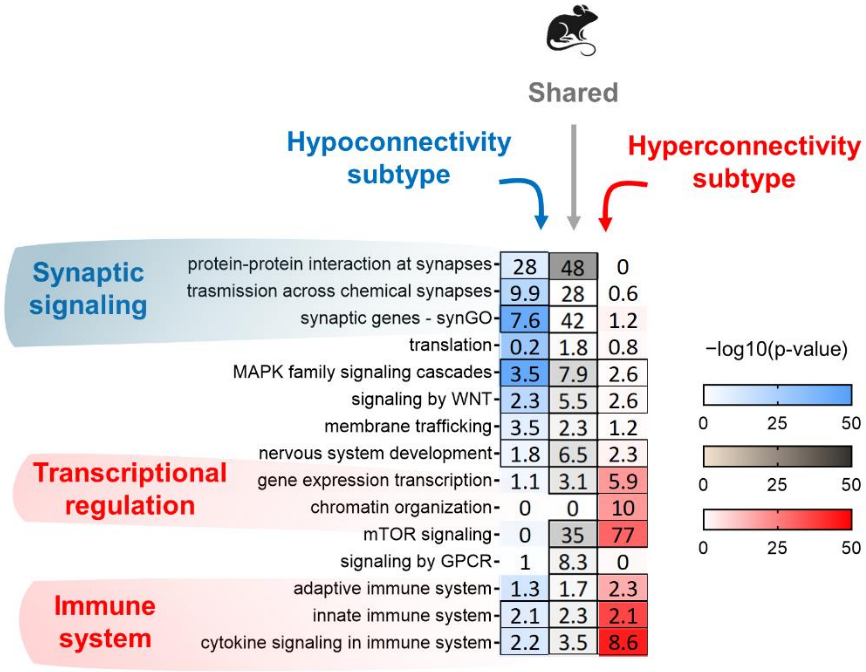

Supplementary Figure 9. Heatmap displaying enrichment for autism-relevant pathways shared by the two interactomes. The odds ratio for genes uniquely part of the interactome of hypoconnectivity subtype are shown in the left column; those shared by both connectivity subtype in the middle column, and those of the hyperconnectivity subtype are in the right column. Thick cell borders indicate that enrichment is significant at q(FDR) < 0.05.

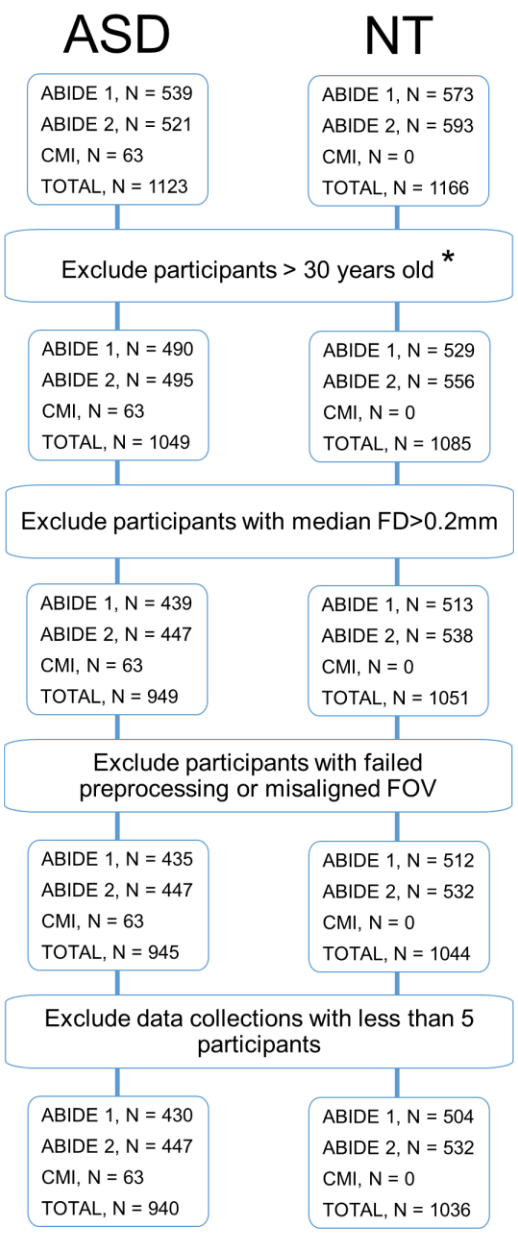

Supplementary Figure 10. Selection flowchart for the human sample. The flowchart illustrates the selection process resulting in the cohort of individuals with a diagnostic label of autism spectrum disorder (N=940 ASD) and neurotypical controls (N=1036 NTs) included in this study. At each flowchart step, we report the number of individual data retained from ABIDEI, ABIDEII repositories and within the collections from CMI as total and by diagnostic group. As a result of this selection process, we examined N=1976 brain scans across 23 data collection MRI sites and 38 data collections, including 18 collections from ABIDEI, 19 collections from ABIDEII and one from CMI. Specifically, ABIDEI included: Caltech (ASD n=14; NT n=13), KKI (ASD n=20; NT n=33), Leuven-1 (ASD n=13; NT n=15), Leuven-2 (ASD n=15, NT n=20), MaxMun (ASD n=11; NT n=20), NYU (ASD n=76; NT n=101), OHSU (ASD n=13, NT n=15), Olin (ASD n=18; NT n=15), Pitt (ASD n=22, NT n=23), SDSU (ASD n=13, NT n=22), Stanford (ASD n=18; NT n=20), Trinity (ASD n=23, NT n=24), UCLA-1 (ASD n=39, NT n=31), UCLA-2 (ASD n=9, NT n=13), UM-1 (ASD n=42, NT n=54), UM-2 (ASD n=13, NT n=21), USM (ASD n=43, NT n=36), Yale (ASD n=28, NT n=28). ABIDEII included: BNI-1 (ASD n=12, NT

n=10), EMC-1 (ASD n=22, NT n=24), ETH-1 (ASD n=8, NT n=22), GU-1 (ASD n=43, NT n=53), IP-1 (ASD n=19, NT n=23), IU-1 (ASD n=16, NT n=17), KKI-1 (ASD n=45, NT n=148), KUL-3 (ASD n=23, NT n=0), NYU-1 (ASD n=47, NT n=30), NYU-2 (ASD n=27, NT n=0), OHSU-1 (ASD n=36, NT n=55), ONRC-2 (ASD n=22, NT n=30), SDSU-1 (ASD n=33, NT n=24), SU-2 (ASD n=20, NT n=20), TCD-1 (ASD n=16, NT n=19), UCD-1 (ASD n=17, NT n=14), UCLA-1 (ASD n=14, NT n=16), U-Mia-1 (ASD n=13, NT n=15), USM-1 (ASD n=14, NT n=12). CMI included ASD n=63 and NT n=0. *Participants >30 years of age were excluded from the analysis as they correspond to less than 5% of the ABIDE repository. FOV, field of view.

Supplementary Table 1

	Hypoconnectivity autism subtype	Hyperconnectivity autism subtype	Group comparisons, statistics and p-values
**Sample size**<br>#	74	162	-
**Data collections***<br>#	28	38	-
**Sex**<br># M, F	59,15	139, 23	$\chi_{(1)}^2$ =1.38,<br>p=0.24,<br>$p_{(FDR)}$=0.61
**Age**<br>years	14.5 (5.4)<br>[7.2-28.0]	13.7 (5.2)<br>[5.6-28.1]	$t_{(234)}$=1.05,<br>p=0.29,<br>$p_{(FDR)}$=0.61
**Full Scale IQ****<br>standard scores	106.9 (18.9)<br>[72-148]	104.5 (16.3)<br>[61-142]	$t_{(199)}$=0.94,<br>p=0.35,<br>$p_{(FDR)}$=0.61
**Verbal IQ*****<br>standard scores	107.7 (18.5)<br>[69-145]	105.3 (16.7)<br>[66-149]	$t_{(175)}$ = 0.86,<br>p = 0.38,<br>$p_{(FDR)}$=0.61
**Non-Verbal IQ******<br>standard scores	104.8 (19.7)<br>[71-157]	103.4 (16.5)<br>[59-146]	$t_{(182)}$ = 0.48,<br>p = 0.63,<br>$p_{(FDR)}$=0.63
**median FD**<br>mm	0.061 (0.04)<br>[0.02-0.18]	0.064 (0.04)<br>[0.01-0.18]	$t_{(234)}$ = 0.62,<br>p = 0.53,<br>$p_{(FDR)}$=0.63
**Psychiatric comorbidity rate^**<br>n(%)	12 (48)	34 (50)	$\chi_{(1)}^2$=0.024,<br>p=0.62,<br>$p_{(FDR)}$=0.63

**Psychoactive medication use ^^** n(%)	47(34)	22(39)	$\chi_{(1)}^2 = 4.04$, $p = 0.04$, $p_{(FDR)}=0.32$

Supplementary Table 1. Demographics and characteristics of the fMRI hypo- and hyperconnectivity autism subtypes. For continuous variables, group mean, and standard deviations are reported in parentheses and minima and maxima are reported in brackets. * The data aggregate of the hypoconnectivity subtype included participants with ASD from all data collections but ABIDEII-ETH-1, ABIDEII-IP-1, ABIDEII-NYU-1, ABIDEII-NYU-2, ABIDEII-UCD-1, ABIDEII-UCLA-1, ABIDEI-MaxMun, ABIDEI-OHSU, ABIDEI-SDSU, ABIDEI-UCLA-2. The data aggregate of the hyperconnectivity subtype included participants with ASD from all n=38 data collections. ** Full Scale IQ was available for n=143 individuals included in the hypoconnectivity subtype and n=58 individuals in the hyperconnectivity subtype. *** Verbal IQ was available for n=124 individuals in the hypoconnectivity subtype and n=53 individuals in the hyperconnectivity subtype. **** Non-Verbal IQ was available for n=128 individuals in the hypoconnectivity subtype and n=56 individuals in the hyperconnectivity subtype. ^ Number and percentage of individuals with one or more psychiatric diagnosis cooccurring with autism. Comorbidity was assessed in a subset of data; specifically, it was assessed in n=27 individuals in the hypoconnectivity subtype and n=68 individuals in the hyperconnectivity subtype. ^^ Number and percentage of individuals using psychoactive medications among the subset of data with information on their use (i.e., n=56 individuals in the hypoconnectivity subtype and n=137 individuals in the hyperconnectivity subtype). M, males; F, females; FD, framewise displacement; χ2, chi-square statistics; t, unpaired t-test statistics.

References

1 Lord, C. *et al.* The autism diagnostic observation schedule-generic: a standard measure of social and communication deficits associated with the spectrum of autism. *J Autism Dev Disord* **30**, 205-223 (2000).

2 Lord, C. *et al. Autism Diagnostic Observation Schedule, Second Edition (ADOS-2).* (Western Psychological Services, 2012).

3 Hus, V. & Lord, C. The autism diagnostic observation schedule, module 4: revised algorithm and standardized severity scores. *J Autism Dev Disord* **44**, 1996-2012 (2014). https://doi.org:10.1007/s10803-014-2080-3

4 Gotham, K., Pickles, A. & Lord, C. Standardizing ADOS scores for a measure of severity in autism spectrum disorders. *J Autism Dev Disord* **39**, 693-705 (2009). https://doi.org:10.1007/s10803-008-0674-3

5 Hus, V., Gotham, K. & Lord, C. Standardizing ADOS Domain Scores: Separating Severity of Social Affect and Restricted and Repetitive Behaviors. *Journal of Autism and Developmental Disorders* **44**, 2400-2412 (2014). https://doi.org:10.1007/s10803-012-1719-1

6 Johnson, W. E., Li, C. & Rabinovic, A. Adjusting batch effects in microarray expression data using empirical Bayes methods. *Biostatistics* **8**, 118-127 (2007). https://doi.org:10.1093/biostatistics/kxj037
