# Supplementary material for: Autism subtypes identified using cross-species functional connectivity analyses
Source: Nat Neurosci. 2026 May 15;29(6):1476–87. doi: 10.1038/s41593-026-02287-z (PMC13246445; doi:10.1038/s41593-026-02287-z)
Supplement: Supplementary file 2 — Reporting Summary [file 41593_2026_2287_MOESM2_ESM.pdf]

nature portfolio

Corresponding author(s): Alessandro Gozzi

Last updated by author(s): 17/02/2026

Reporting Summary

Nature Portfolio wishes to improve the reproducibility of the work that we publish. This form provides structure for consistency and transparency in reporting. For further information on Nature Portfolio policies, see our Editorial Policies and the Editorial Policy Checklist.

Statistics

For all statistical analyses, confirm that the following items are present in the figure legend, table legend, main text, or Methods section.

n/a	Confirmed	
☐	☒	The exact sample size (*n*) for each experimental group/condition, given as a discrete number and unit of measurement
☐	☒	A statement on whether measurements were taken from distinct samples or whether the same sample was measured repeatedly
☐	☒	The statistical test(s) used AND whether they are one- or two-sided<br>*Only common tests should be described solely by name; describe more complex techniques in the Methods section.*
☐	☒	A description of all covariates tested
☐	☒	A description of any assumptions or corrections, such as tests of normality and adjustment for multiple comparisons
☐	☒	A full description of the statistical parameters including central tendency (e.g. means) or other basic estimates (e.g. regression coefficient) AND variation (e.g. standard deviation) or associated estimates of uncertainty (e.g. confidence intervals)
☐	☒	For null hypothesis testing, the test statistic (e.g. *F*, *t*, *r*) with confidence intervals, effect sizes, degrees of freedom and *P* value noted<br>*Give P values as exact values whenever suitable.*
☐	☒	For Bayesian analysis, information on the choice of priors and Markov chain Monte Carlo settings
☐	☒	For hierarchical and complex designs, identification of the appropriate level for tests and full reporting of outcomes
☐	☒	Estimates of effect sizes (e.g. Cohen's *d*, Pearson's *r*), indicating how they were calculated

Our web collection on statistics for biologists contains articles on many of the points above.

Software and code

Policy information about availability of computer code

Data collection	Mouse MRI scans were acquired with two 7T Buker Biospin with Bruker Paravision Software (v6). Details of data collection for human fMRI scans are available at at https://fcon_1000.projects.nitrc.org/indi/abide/abide_I.html and https://fcon_1000.projects.nitrc.org/indi/abide/abide_II.html. Source data for figures are provided with this paper.
Data analysis	Mouse MRI scans were analyzed with FSL (v6.0), AFNI (v21.0) and ANTS (v2.1), python (v3) and GraphPad (v9.2). Whole brain network modelling was carried out with python (v3). Human MRI scans were analyzed with C-PAC (v6.0), python (v3) and GraphPad (v9.2). Gene enrichment analysis was carried out with R (v4.1). Behavioral scores were analyzed with GraphPad (v9.2). The code used for preprocessing mouse fMRI data is available at https://github.com/functional-neuroimaging/rsfMRI-preprocessing. The code for mapping global connectivity in mice and humans is available at https://github.com/functional-neuroimaging/rsfMRI-global-local-connectivity. The code employed for gene enrichment analysis is available at https://github.com/functional-neuroimaging/gene_decoding_and_enrichment.The code for site harmonization and cluster analysis is available at: https://github.com/functional-neuroimaging/biological_subtypingThe authors also declare that gene expression data are publicly available in the web portal of the Allen Brain Institute (http://www.brain-map.org/).

For manuscripts utilizing custom algorithms or software that are central to the research but not yet described in published literature, software must be made available to editors and reviewers. We strongly encourage code deposition in a community repository (e.g. GitHub). See the Nature Portfolio guidelines for submitting code & software for further information.

Data

Policy information about availability of data

All manuscripts must include a data availability statement. This statement should provide the following information, where applicable:

- Accession codes, unique identifiers, or web links for publicly available datasets
- A description of any restrictions on data availability
- For clinical datasets or third party data, please ensure that the statement adheres to our policy

Raw mouse fMRI timeseries can be download at: https://dataverse.iit.it/. The link will be made openly accessible upon acceptance of the manuscript. R1) I updated the information under data including the statement that follows Regarding the human-related data, those selected from ABIDE I and II are available at https://fcon_1000.projects.nitrc.org/indi/abide/abide_I.html and https://fcon_1000.projects.nitrc.org/indi/abide/abide_II.html; most of those included in the CMI-based dataset are deposited in the National Database for Autism Research (NDAR; collection DOI: 10.15154/nnfr-4943) upon parent/legal guardian consent and are accessible through NDAR (https://ndar.nih.gov/) in accordance with their data use policies.

Research involving human participants, their data, or biological material

Policy information about studies with human participants or human data. See also policy information about sex, gender (identity/presentation), and sexual orientation and race, ethnicity and racism.

Reporting on sex and gender	We reported biological sex in the manuscript
Reporting on race, ethnicity, or other socially relevant groupings	This information is not reported in the manuscript
Population characteristics	Key demographic and behavioral characteristics have been reported in the manuscript
Recruitment	Participants in the CMI cohort were recruited in the new York metropolitan area according to local site procedures, as described in related publications referenced in the manuscript (e.g., Segura et al 2025; Simhal et al. 2021). No additional recruitment was performed for the present study, which analyzed de-identified data. ABIDE I/II participants were recruited at their respective contributing sites as part of the original studies.
Ethics oversight	Each data contributing site obtained ethical approval, and informed consent and/or assent were obtained in accordance with their local IRB/ethics committee.

Note that full information on the approval of the study protocol must also be provided in the manuscript.

Field-specific reporting

Please select the one below that is the best fit for your research. If you are not sure, read the appropriate sections before making your selection.

☒ Life sciences ☐ Behavioural & social sciences ☐ Ecological, evolutionary & environmental sciences

For a reference copy of the document with all sections, see nature.com/documents/nr-reporting-summary-flat.pdf

Life sciences study design

All studies must disclose on these points even when the disclosure is negative.

Sample size	Sample size is reported in the figure caption for each experiment. No statistical methods were used to predetermine sample sizes for our experiments, but our sample sizes are comparable or higher to those reported in previous publications on mouse rsfMRI (Liska et al. 2018, Pagani et al. 2019, Bertero et al. 2018, Sutterlin et al. 2018, Sforazzini et al. 2016), dMRI (Liska et al. 2018, Pagani et al. 2019, Sforazzini et al. 2016), behavior (Liska et al. 2018, Pagani et al. 2019) and human neuroimaging studies on ASD (Hong et al. 2019, Superkar et al. 2013).
Data exclusions	We excluded participants aged 30 or older, with high in scanner head motion (FD>0.2mm), with misalligned FOV or those on sites (collections) with n equal 5 or lower following these criteria . No mouse data were excluded from this study.
Replication	Replication analysis was conducted for the human fMRI study. Replication was quantified with Dice coefficients and Pearson's spatial correlation. The complex and lengthy design of our mouse studies did not allow us to perform replication studies, as such endeavor would also have important ethical implications in terms of 3R compliance. We however thoroughly randomized genotype and treatment groups, making analysts blind to treatment genotype. This strategy, together with the statistical robustness of our experimental findings, suggest our results would be reproducible across repetition.
Randomization	For all mouse experiments, animals were chosen based on genotypes. Aged-matched wild-type and mutant littermates were compared to minimize variance in age, genetic background and environment. For human data, individuals were grouped by diagnosis (ASD vs. control population)
Blinding	All data acquisition and analysis were performed blind to the genotype/treatment condition.

Reporting for specific materials, systems and methods

We require information from authors about some types of materials, experimental systems and methods used in many studies. Here, indicate whether each material, system or method listed is relevant to your study. If you are not sure if a list item applies to your research, read the appropriate section before selecting a response.

Materials & experimental systems

n/a	Involved in the study	
☒	☐	Antibodies
☒	☐	Eukaryotic cell lines
☒	☐	Palaeontology and archaeology
☐	☒	Animals and other organisms
☐	☒	Clinical data
☒	☐	Dual use research of concern
☒	☐	Plants

Methods

n/a	Involved in the study	
☒	☐	ChIP-seq
☒	☐	Flow cytometry
☐	☒	MRI-based neuroimaging

Animals and other research organisms

Policy information about studies involving animals; ARRIVE guidelines recommended for reporting animal research, and Sex and Gender in Research

Laboratory animals	We used laboratory mice (mus musculus). A complete list of the mice used with genotypes, sex, age and corresponding references is reported in Supplementary table 1.
Wild animals	No wild animals were used in the study
Reporting on sex	We carried out analysis for both sexes, n=123 females and n=426 males
Field-collected samples	This study did not involve field collected samples.
Ethics oversight	Animal studies were conducted in accordance with the Italian Law (DL 26/2014, EU 63/2010, Ministero della Sanità, Roma) and the recommendations in the Guide for the Care and Use of Laboratory Animals of the National Institutes of Health. Animal research protocols were reviewed and consented to by the animal care committee of the Istituto Italiano di Tecnologia and the Italian Ministry of Health. All surgical procedures were performed under anesthesia.

Note that full information on the approval of the study protocol must also be provided in the manuscript.

Clinical data

Policy information about clinical studies

All manuscripts should comply with the ICMJE guidelines for publication of clinical research and a completed CONSORT checklist must be included with all submissions.

Clinical trial registration	NA
Study protocol	NA
Data collection	NA
Outcomes	We used ADOS as the behavioral outcome for symptoms severity.

Plants

Seed stocks	NA
Novel plant genotypes	NA
Authentication	NA

Magnetic resonance imaging

Experimental design

Design type	Resting state fMRI for mice and humans.
Design specifications	Resting state fMRI for mice and humans.
Behavioral performance measures	We used ADOS scores as the behavioral outcome for symptoms severity, other behvaioral meausres have been used to characterize the sample

Acquisition

Imaging type(s)	Functional
Field strength	7T for animals. For humans see the field strength used in each laboratory in the publicly available repository http://fcon_1000.projects.nitrc.org/indi/abide
Sequence & imaging parameters	Mouse rsfMRI: Functional images were acquired with a 7T MRI scanner (Bruker Biospin, Milan – Bruker Paravision v6) and using a 72-mm birdcage transmit coil and a 4-channel solenoid coil for signal reception (Liska et al., 2015). For each session, in-vivo anatomical images were acquired with a fast spin echo sequence (repetition time [TR] = 5500 ms, echo time [TE] = 60 ms, matrix 192 × 192, field of view 2 × 2 cm, 24 coronal slices, slice thickness 500 μm). Co-centered single-shot BOLD rsfMRI time series were acquired using an echo planar imaging (EPI) sequence with the following parameters: TR/TE = 1000/15 ms, flip angle 30°, matrix 100 × 100, field of view 2.3 × 2.3 cm, 18 coronal slices, slice thickness 600 μm for 1920 volumes.<br><br>For humans see sequence and imaging parameters used in each laboratory in the publicly available repository http://fcon_1000.projects.nitrc.org/indi/abide
Area of acquisition	Whole brain imaging for both mice and humans

Diffusion MRI ☐ Used ☒ Not used

Preprocessing

Preprocessing software	Mouse rsfMRI: removal of first 50 volumes (AFNI v.21 3dTcat), despiking (AFNI 3dDespike), motion correction (FSL v.6 mcflirt), skull-stripping (FSL bet), spatial normalization with affine and diffeomorphic registration (ANTS v.2 antsRegistration + antsApplyTransforms) to a skull-stripped reference BOLD template, calculation of motion traces of head realignment parameters (3 translations + 3 rotations) and mean ventricular signal (corresponding to the averaged BOLD signal within a reference ventricular mask, FSL, fslmeants), regression of nuisance parameters (FSL, fsl_regfilt), band-pass filtering (AFNI 3dBandpass) and spatial smoothing (AFNI, 3dBlurInMask).<br><br>Human rsfMRI: Preprocessing and denoising of participants with and without autism has been carried out with C- PAC
Normalization	Mouse rsfMRI: data was registered with a combination of affine and non-linear transformations using FSL's FLIRT<br>Human rsfMRI: data underwent affine co-registration to structural image and then nonlinear warping to MNI space using C-PAC
Normalization template	Mouse rsfMRI: In-house EPI mouse brain template available at https://github.com/functional-neuroimaging/rsfMRI-templates<br>Human rsMRI: MNI space 2 mm isotropic
Noise and artifact removal	Mouse rsfMRI: Nuisance variables of 6 head realignment parameters and mean ventricular signal. Band-pass filtering 0.01-0.1Hz.<br><br>Human rsfMRI: Nuisance variables of head motion and mean ventricular signal plus CompCorr of white matter signal.(30 parameters). Band-pass filtering 0.01-0.1 Hz.
Volume censoring	Not performed

Statistical modeling & inference

Model type and settings	Mouse rsfMRI: Cohen's d<br><br>Human rsfMRI: t-test
Effect(s) tested	Mouse rsfMRI: Pearson's correlation, Cohen's d for effect size<br><br>Human rsfMRI: Pearson's correlation, multiple unpaired t-tests FDR-corrected

Specify type of analysis: ☒ Whole brain ☐ ROI-based ☐ Both

Statistic type for inference<br>(See Eklund et al. 2016)	Mouse rsfMRI: Based on intergroup differences of spatially unbiased global connectivity mapping<br><br>Human rsfMRI: Based on intergroup differences of spatially unbiased global connectivity mapping
Correction	Voxelwise threshold: |t| > 3.1 and family-wise error (FWER) cluster-corrected using a cluster threshold of p < 0.01

Models & analysis

n/a	Involved in the study
☐	☒ Functional and/or effective connectivity
☐	☒ Graph analysis
☐	☐ Multivariate modeling or predictive analysis

Functional and/or effective connectivity	Pearson's correlation
Graph analysis	Unbinarised global connectivity
Multivariate modeling and predictive analysis	Mouse rsfMRI: genotype<br><br>Human rsfMRI: diagnosis (ASD vs. CTR)
